# Theranostic nanoparticles for the management of thrombosis

**DOI:** 10.7150/thno.70001

**Published:** 2022-03-14

**Authors:** Peije Russell, Christoph Eugen Hagemeyer, Lars Esser, Nicolas Hans Voelcker

**Affiliations:** 1 Drug Delivery, Disposition & Dynamics, Monash Institute of Pharmaceutical Sciences, Monash University, Parkville, Victoria, 3052, Australia.; 2 NanoBiotechnology Group, Australian Centre for Blood Diseases, Central Clinical School, Monash University, Melbourne, Victoria, 3004, Australia.; 3 Commonwealth Scientific and Industrial Research Organisation (CSIRO), Manufacturing, Clayton, Victoria, 3168, Australia.; 4 Melbourne Centre for Nanofabrication, Victorian Node of Australian National Fabrication Facility, Clayton, Victoria, 3168, Australia.; 5 Department of Materials Science and Engineering, Monash University, Clayton, Victoria, 3800, Australia.

**Keywords:** Thrombosis, Theranostics, Nanomedicine, Diagnostic imaging, Thrombolytic therapy

## Abstract

Acute thrombosis and thromboembolisms are one of the leading causes of mortality and morbidity in both developed and developing countries, placing a huge burden on health and economic systems. Early diagnosis is critical but currently limited in accuracy and hampered by a narrow time frame, where the short therapeutic window also severely restricts treatment options. Additionally, clinically used antithrombotics and thrombolytics suffer from severe side effects and are limited in efficacy by a short half-life and susceptibility to degradation. The use of systems containing both diagnostic and therapeutic moieties, known as theranostics, can potentially improve patient outcomes by increasing the precision and efficacy of diagnosis and treatment, enabling personalised and precision medicine. Leveraging nanomedicine may further improve treatment by improving the system's pharmacokinetic properties including controlled drug delivery. This review provides an overview of the development of such theranostic nanoparticle systems, with a focus on approaches that may be utilised to usher this field towards clinical use.

## Introduction

Cardiovascular disease (CVD) is the leading cause of mortality, with a prevalence of 523.2 million cases and 18.6 million deaths globally in 2019 alone. With an estimated cost of $363.4 billion annually in the United States alone, CVD has a truly enormous socioeconomic impact. The prevalence and mortality of CVD have risen significantly since 2010, and are expected to continue rising 1. Thrombosis, or the excessive formation of blood clots, is a leading contributor to CVD. These thrombi can cause an obstruction of blood flow at the site of origin (acute thrombosis) or dislodge and become an embolus stopping flow at a distal site (thromboembolism). Both forms of blood clots may lead to different manifestations of catastrophic events including stroke, myocardial infarction, pulmonary embolism, and deep vein thrombosis 2. Such events have a high chance of causing mortality, and long-term morbidity is commonly experienced by survivors. Therefore, there is an ever-growing need to improve treatment outcomes of thrombosis.

### Thrombosis pathophysiology

Traditionally, thrombotic events are closely related to CVD and associated lifestyle choices, such as diet, exercise regimes, and smoking 1. However, recently a new cause of thrombosis has gained considerable attention, with extensive reports of COVID-19 related thrombosis 3, 4. Additionally, the AstraZeneca (ChAdOx1-S) and Johnson & Johnson (AD26.COV2.S) adenoviral COVID-19 vaccines have been shown to cause venous thromboembolic events as a rare side effect 5. Reports of such side effects causing serious injury and death have resulted in vaccinations being temporarily suspended in certain countries, hampering immunisation efforts 6. This further highlights the need for improved diagnosis and treatment of thrombosis, as this would aid greatly in the efforts of preventing COVID-19 related deaths and long-term injury.

**Figure 1:**
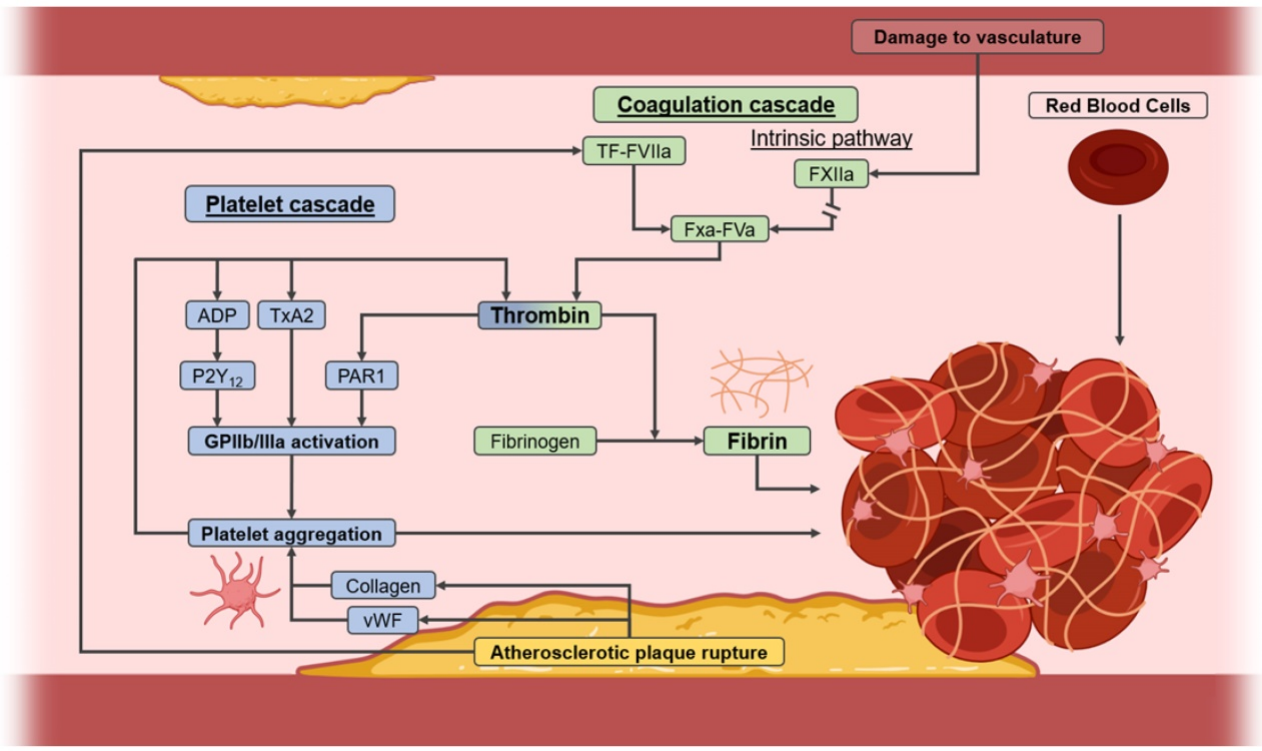
** Pathways towards the formation of a thrombus as a result of atherosclerotic plaque rupture.** Collagen, von Willebrand factor (vWF), and tissue factor (TF) are exposed to plasma upon rupture of an atherosclerotic plaque, resulting in activation of the platelet and the coagulation cascade. Platelet aggregation stimulates the release of platelet-activating factors, resulting in a self-amplifying platelet cascade. Thrombin activates platelets and cleaves fibrinogen to produce fibrin, which binds to the activated platelets, entrapping red blood cells and forming the thrombus.

Most commonly however, thrombi are formed upon rupture of unstable atherosclerotic plaques, which are often present as a result of hyperlipidaemia, obesity, smoking, or diabetes 1. Upon rupture of a plaque, the blood is exposed to procoagulant components, which cause platelets, fibrin, and red blood cells (RBCs) to combine to form the thrombi 2. Two pathways contribute to this process known as thrombogenesis, namely the coagulation cascade and the platelet cascade (**Figure 1**). Of particular interest is the production of fibrin from its inactive form - fibrinogen - through cleavage by thrombin. Thrombin also indirectly activates glycoprotein IIb/IIIa on platelets, and is thus involved in both cascades. Activated glycoprotein IIb/IIIa then binds fibrin, after which the bound platelets release secondary messengers, which includes a burst of thrombin, further accelerating the growth of the thrombus. Hence, due to their extensive involvement in both cascades, fibrin, thrombin, and activated glycoprotein IIb/IIIa on platelets are all prominent targets of interest in the pathophysiology of thrombosis 2.

Notably, the relative amounts of the beforementioned constituents vary depending on the origin of the thrombus, as recently shown by Chernysh *et al.* 7. Arterial thrombi, as formed in the high-shear environment of an artery, contain roughly 31% platelets, whereas venous thrombi contain only 0.4%. This high platelet-content gives arterial thrombi their distinct white appearance. The lack of platelets in venous thrombi is compensated for with a larger amount of RBCs, resulting in their red appearance 7. Due to these differences in their structure and origin, arterial and venous thrombi may need to be considered as separate targets. Despite these differences, all thrombi also have certain aspects in common. For instance, all thrombi contain a comparable amount of fibrin (35-43%), making this a target of interest for therapeutics. Additionally, all thrombi contain little space between their structural components, making them very dense 7. This makes the thrombi harder to break down and limits penetration of therapeutics, further indicating the need for improved therapeutic approaches.

### Current imaging diagnosis and treatment

If a patient is at risk of thrombosis, or a thromboembolism is suspected to have already occurred based on symptoms described by the patient, imaging is commonly the next step to confirm such a diagnosis. Particularly in acute thromboembolisms, an accurate diagnosis is vital to ensure the patient is treated correctly. It should be noted that, based on the type of thrombosis, diagnosis and treatment options can vary widely, hence the location and type of thrombosis is a major factor in choosing the correct approach. For instance, deep vein thrombosis (DVT) occurs in the legs and is diagnosed through compression ultrasound (US), where the compression of veins and obstruction of blood flow are used as indications of the presence of a thrombus 8. This technique has a high sensitivity for DVT above the knee (96.5%), but has a lower sensitivity for DVT below the knee (71.2%) 9. Pulmonary embolism and stroke are primarily diagnosed using computed tomography (CT) and magnetic resonance imaging (MRI) 10, 11. CT can quickly identify blood clots but requires ionising radiation and the use of potentially toxic contrast agents. MRI omits these downsides, but has a lower availability due to cost, and requires a much longer imaging time thus extending the time of diagnosis 10, 11. Upon occlusion of the blood vessel, damage to surrounding tissue is rapid and worsens over time 12. Thus, there is a need to identify the blockage as fast as possible and minimise damage. Additionally, the techniques described above lack the ability to detect thrombi in the early stages of thrombosis, as they can only detect large blood clots and their effect on blood flow after embolism. This prevents the early detection of thrombi and thus the prevention of catastrophic events such as stroke and pulmonary embolisms. Therefore, diagnostic techniques require improvement for earlier and faster detection of thrombosis.

Antithrombotic treatment also depends greatly on the type of the thrombus, where three classes of therapeutics are commonly used for prophylaxis and acute treatment. Anticoagulants, such as heparins and vitamin K antagonists (e.g. warfarin), inhibit the coagulation cascade by inhibiting FXa and/or thrombin to prevent the generation of fibrin 11. On the other hand, antiplatelet agents, such as aspirin and cangrelor, inhibit the platelet cascade by inhibiting the activation of glycoprotein IIb/IIIa through various approaches. As venous thrombi lack platelets, antiplatelet drugs are primarily used for the treatment of arterial thrombi. Both antiplatelets and anticoagulants are available as oral and injectable therapeutics, where oral therapeutics are used for the prevention of thrombosis, and intravenous injections may be performed in the case of acute thrombotic events 2.

Severe thrombotic events, such as ischemic stroke, myocardial infarction, or massive pulmonary embolism, are treated with thrombolytic therapeutics, mechanical thrombectomy, or percutaneous transluminal coronary angioplasty (PTCA). Mechanical thrombectomy and PTCA involve the removal of thrombi through surgical interventions, which remain invasive despite ongoing technological advancements 13. Therefore, administration of thrombolytics, such as streptokinase or tissue plasminogen activator (tPA), is often preferred. These therapeutics stimulate activation of plasmin, which cleaves fibrin to degrade the thrombus. Thrombolytics can also be used for less acute events (subacute and/or chronic), though the time windows for such treatment vary. US facilitated catheter-directed thrombolysis may also be performed, though this increases the cost and invasiveness of the procedure 14. The main determinant for the choice of treatment, however, is the age of the thrombus, where older (“chronic”) thrombi become increasingly resistant to thrombolytic therapy, hence mechanical thrombectomy and PTCA are utilised instead in such cases 15.

Despite these limitations, all therapeutics described have high efficacy if applied correctly. However, their use is limited considerably by their severe side effects. As all therapeutics promote the breakdown or prevention of thrombi through activation of naturally present systems, the haemostatic system becomes unbalanced. Where platelet formation and fibrin generation are naturally used to cause blood clotting upon injury to blood vessels, inhibition of these processes can lead to severe bleeding complications 16, 17. This severely limits their application, particularly in patients with an increased bleeding risk. Additionally, as most thrombotic therapeutics are proteins, they are susceptible to degradation *in vivo,* lowering their efficacy. Thus, further improvements in the treatment of thrombosis are required.

### Theranostics and nanoparticles

Improvement of both the diagnosis and treatment of thrombosis can be made through the development of theranostics. The term “theranostics”, as first coined by Funkhouser, describes the combined delivery of imaging and therapeutic modalities, enabling tracking of the therapeutic after administration 18. Diagnosis of the disease may be performed, while treating the patient, enabling a much faster response to thrombotic events. It also allows for fast, individualised alterations to dosage to be made, resulting in a personalised or precision medicine approach.

Though not yet clinically available for the treatment of thrombosis, theranostics have already made large impacts in the clinical setting for the treatment of other diseases. A prominent example is the use of radioiodine in the treatment of thyroid cancer. ^131^I is actively taken up by the overactive thyroid gland, emitting both β and γ radiation, and can therefore be used to induce apoptosis in tumour cells while simultaneously being tracked through single-photon emission computed tomography (SPECT). This simple yet effective theranostic is used extensively 19; showing the potential of clinical translation of theranostics, particularly those that may require little extra effort compared to traditional therapeutics.

Historically, the combination of thrombosis therapeutic and diagnostic moieties has mostly been performed in the form of radiolabelling of thrombolytics to determine biodistribution and efficacy profiles. Examples include ^123^I-labelled urokinase and radiolabelling of urokinase and streptokinase with ^131^I or ^99m^Tc 20, 21. Though at the time the concept of theranostics was far from adoption, these systems may still be considered as such, as modern-day SPECT scanners may be able to track the distribution and subsequent thrombolysis caused by these systems. Unfortunately, these systems would be unlikely to see clinical use, as they fail to improve upon the main limitation exhibited by thrombolytics: their severe side effects.

To improve upon therapeutics, incorporation into nanomedicine has gained widespread interest. The development of theranostic nanoparticle systems enables benefits such as increased circulation time and decreased side effects due to reduced renal clearance and physical shielding from non-target tissues. Additional functionality, such as active targeting and responsive drug release can further improve the distribution and pharmacokinetic profile. Nanoparticle systems have previously successfully been utilised in clinical settings for other diseases, most prominently the recent Pfizer-BioNTech COVID-19 vaccine 22. Nanoparticles for theranostic purposes are yet to reach the market, though clinical trials are being performed, such as Crystal Therapeutic's Phase I trial of [^89^Zr]-Df-CriPec^®^ docetaxel, an ^89^Zr-labelled nanoparticle system delivering docetaxel for treatment of solid tumours 23.

### Scope of this review

Despite the promising characteristics of theranostic nanoparticles for thrombosis management, no such systems are currently in clinical use. Therefore, this review aims to summarise the current literature on such theranostic systems, and provide a perspective on how to increase the clinical potential of this field. Where other reviews have focussed on the use of nanoparticles solely for the delivery of drugs for the treatment of thrombosis 24, 25, a theranostic approach requires a far more sophisticated design, where the simultaneous delivery of diagnostic and therapeutic introduces novel challenges. Several reviews on theranostic nanoparticles for CVD have correctly identified this issue 26, 27, but the broad scope of these reviews limits them from providing an in-depth exploration of the application of theranostic nanoparticles towards thrombosis. Therefore, this review focuses on recent advancements and challenges specifically in the field of theranostic nanoparticles for thrombosis, and how to overcome said challenges.

**Figure 2:**
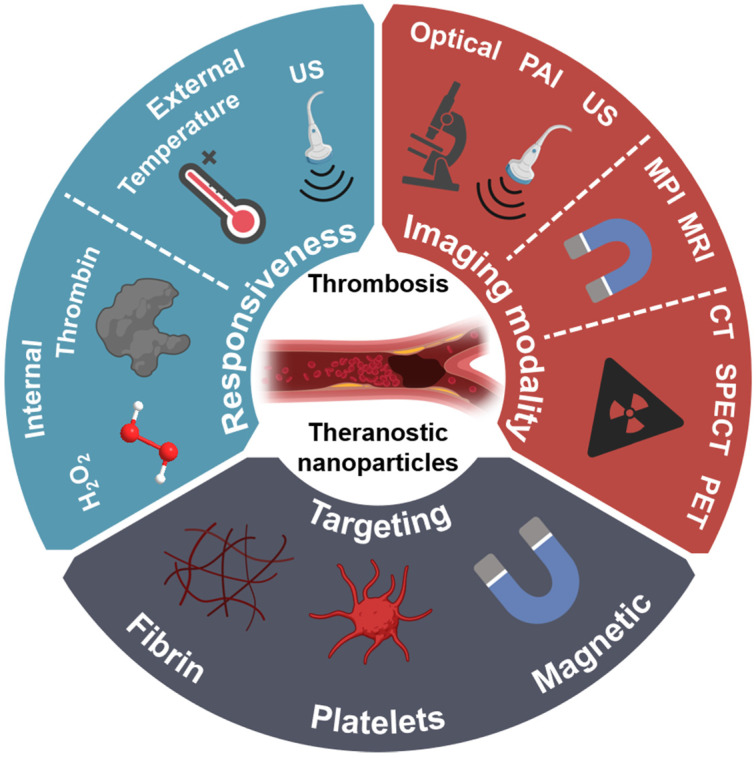
Schematic illustration of the various approaches towards theranostic nanoparticles for diagnosis and treatment of thrombosis.

To maximise the clinical potential of theranostic nanoparticles for thrombosis, several aspects need to be optimised. This includes; (1) safety; (2) simplicity and rapidity; (3) applicability and availability; (4) cost-effectiveness. Therefore, this review provides an in-depth overview of recently published works in this field and assesses their translational potential based on these criteria. Additionally, this review draws from promising approaches not yet utilised in the field of theranostics for thrombosis and provides suggestions on how these may be utilised to elevate the field towards clinical adoption. This includes approaches utilised in drug delivery systems and imaging probes for thrombosis with encouraging results. The utilised and yet-to-be-utilised approaches are divided into three design aspects of theranostic nanoparticles, namely their choice of imaging modality, responsiveness, and active targeting (**Figure 2**). It should be noted that the design of a theranostic nanoparticle system should take a holistic approach, considering the combination of all three approaches to maximise its clinical potential. The use of theranostic nanoparticles for the early detection of thrombosis is also discussed as a highly promising area of research. Finally, the pre-clinical assessment of theranostic nanoparticles for thrombosis is examined, to enable accurate identification of promising leads for clinical applications.

## Theranostic nanoparticles for thrombosis management

A wide range of theranostic nanoparticles for the diagnosis, prevention, and treatment of thrombosis has been developed, containing various imaging moieties and therapeutics (**Table 1**). However, several interesting trends are apparent, where similar approaches have been utilised. One example is the prevalence of iron oxide nanoparticles (IONPs), consistent with the translational potential that these particles have shown in other disease areas. Several IONPs are available on the market, for instance, Ferumoxytol utilises IONPs to treat anaemia in patients with chronic kidney disease 28. The interest in IONPs for theranostics is mostly due to their inherent functioning as an MRI contrast agent, enabling imaging capabilities in these nanoparticle formulations without further modifications. Several of these formulations, such as Feridex and Resovist, have been clinically approved, though they have also subsequently been retracted from the market 29.

Thus, several systems utilising IONPs have been developed as theranostics for thrombosis. By utilising these dual-functional IONPs, very simple and likely cost-effective systems were produced, with excellent imaging capabilities. For example, Groult *et al.* used IONPs to deliver heparin as an anticoagulant, while McCarthy *et al.* delivered tissue plasminogen activator (tPA) for retrospective treatment 30, 31. Unfortunately, these theranostic systems rely on surface loading, which limits the loading capacity and exposes the therapeutic to interactions with off-target tissues and proteolysis by systemic enzymes. This reduces their efficacy and may result in haemorrhagic risk, thus potentially introducing a safety concern. Therefore, more advanced systems have been explored in which IONPs were incorporated in a larger nanoparticle 32-35. This ensures core loading of the therapeutic, circumventing the issues faced by the surface-loaded IONPs, while retaining the MRI contrast abilities of the IONPs. However, this does increase the complexity of the fabrication, reducing the simplicity and cost-effectiveness, and subsequently their potential for clinical applications.

**Figure 3:**
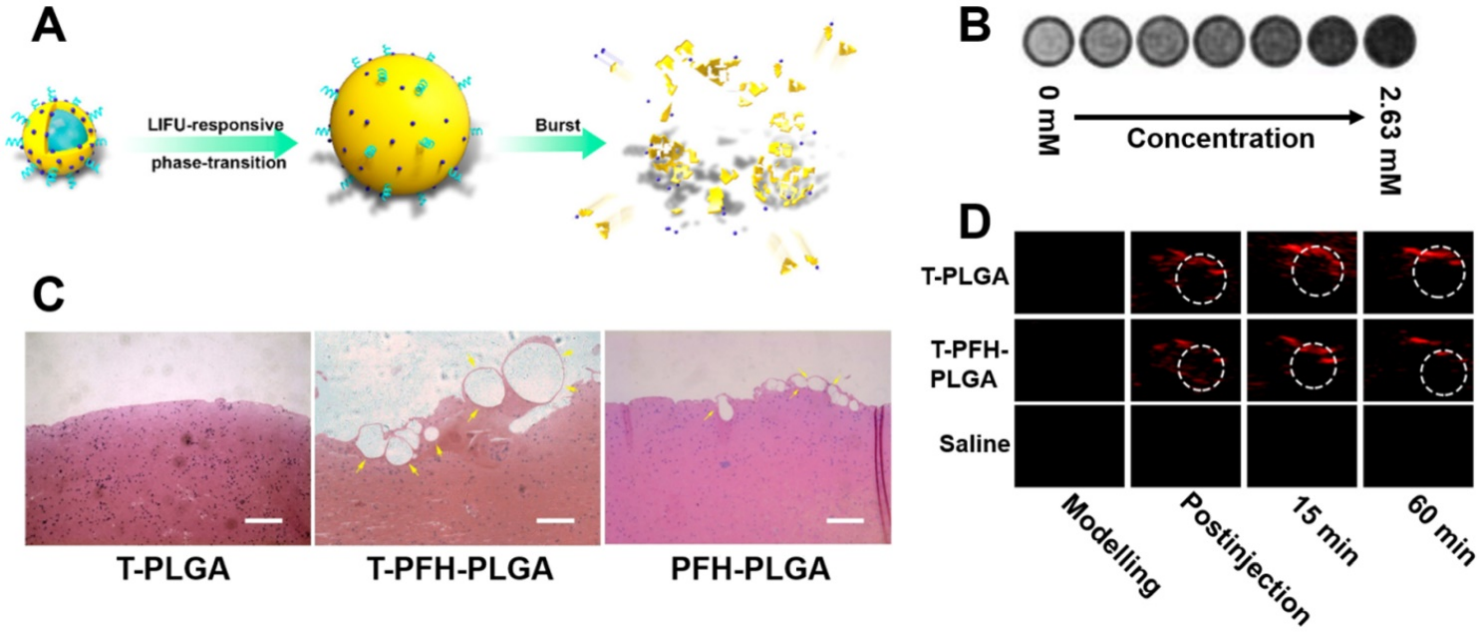
** Fibrin targeting low intensity focused ultrasound (LIFU)-responsive nanodroplet based on a PLGA nanoparticle containing perfluorohexane (PFH), CREKA peptides, and IONPs. A.** Schematic illustration of the focused US responsive behaviour of the theranostic nanoparticles. **B.** MRI analysis of theranostic at various concentrations, indicating its performance as a negative contrast agent. **C.** H&E staining of blood clots after 2 hours of exposure to focused US and PFH-free nanoparticles (T-PLGA), PFH-containing nanoparticles (T-PFH-PLGA) or non-targeted nanoparticles (PFH-PLGA). **D.** PAI images of the abdominal aorta (area marked by the dotted line, axial view) before and after injection of T-PLGA, T-PFH-PLGA and saline. Adapted with permission from Zhong *et al.* 34, copyright 2019 American Chemical Society.

Another approach of interest is the use of switchable nanodroplets, which can turn into microbubbles. These switchable nanodroplets (also known as nanoexcavators or nanobombs) contain a perfluorocarbon (PFC) such as perfluorohexane (PFH) or perfluoropentane (PFP) which undergoes a phase transition to gas upon irradiation with low-intensity focused ultrasound (LIFU), producing a microbubble. Clinical trials have shown that such microbubbles may enhance the clot-degrading activity of a thrombolytic agent upon stimulation with US if co-administered 36, 37. Microbubbles are also known to cause thrombolysis themselves through an effect known as “cavitation”, which involves a microbubble explosion destroying the clot. This was demonstrated by Zhong *et al.* with *ex vivo* imaging of thrombi in a rabbit model after treatment with their nanodroplet system (**Figure 3A**), as shown in **Figure 3C** 34. Additionally, these microbubbles can also enhance penetration into the clot, further improving thrombolysis 34. Simultaneously, several other groups have shown that such microbubbles could enhance US signals, thus adding imaging functionality to the particle and resulting in a simple theranostic system 38, 39. As these particles only become microbubbles upon stimulation with LIFU, both imaging and therapeutic functioning can be turned on specifically at the site of a thrombus 40, 41. However, Zhong *et al.* even went a step further, with the incorporation of IONPs into these particles enabling MRI (**Figure 3B**) and photoacoustic imaging capabilities (**Figure 3D**) as well 34. Though this multimodality increases their range of applications, it also increases the complexity of their fabrication. Therefore, the simpler nanodroplets solely relying on US imaging may be more clinically viable due to their superior cost-effectiveness. However, all these systems are unfortunately limited by their safety profile, as the cavitation they cause has the potential to result in tissue damage 39.

Another interesting approach towards the development of theranostic nanoparticles is the use of cell-derived membranes. By utilising such endogenous-derived materials, the cytotoxicity of the nanoparticulate systems may be reduced 35, 42. Additionally, the presence of “self-marker” proteins on some of these systems enables exceptionally long circulation time in the range of 90-120 days, which would be highly beneficial for the preventative use of antithrombotics 43. Also of particular interest for the treatment of thrombosis is the use of membranes derived from cells naturally present in thrombi, such as red blood cells and platelets, as this enables targeting of thrombi 42, 43. For instance, Xu *et al.* showed they could obtain platelet-derived nanoparticles which displayed cell adhesion proteins without retaining thrombosis-inducing characteristics of the platelets. Thus, this system could be used for effective targeted thrombolysis 42. However, these systems currently rely on optical imaging which has a limited depth of penetration and thus severely limited clinical relevance. The thrombolytic payload is also conjugated to the surface, reducing loading capacity and exposing the thrombolytic to degradation by systemic proteolytic enzymes. Thus, a switch to more clinically relevant imaging and internal loading would greatly benefit their translational potential. Additionally, further research into overcoming challenges such as complex manufacturing may be advantageous.

Despite all these promising developments and applications of nanotechnologies, no theranostic nanoparticles for thrombosis have reached the market yet. As outlined above, current theranostic nanoparticles are limited by the safety profiles of their nanoparticles, the clinical relevance of their imaging modalities, and their complexity of fabrication. However, it is expected that with further development of the mentioned techniques, and with the application of novel techniques, these issues can be overcome to produce clinically viable theranostic nanoparticles for thrombosis.

## Imaging of theranostic nanoparticles

One of the most important considerations when designing a novel theranostic agent is the intended imaging technique. In the case of thromboembolisms, the speed and specificity for the detection of thrombi are especially critical in preventing mortality and morbidity. As previously mentioned, the type of treatment applied relies greatly on the type of thrombosis, hence a rapid and - most importantly - accurate diagnosis is vital to ensure the correct treatment may be applied as soon as possible. Clinically used diagnostic techniques are somewhat lacking in these areas, and emerging techniques may provide improvements. When designing a theranostic nanoparticle, one should consider the ease of incorporation into a theranostic system, invasiveness of the procedure, and, perhaps most importantly, the suitability for the disease state. The imaging technique should be appropriate to the type of thrombosis, where the choice of imaging modality depends on the location, origin, and acuity of thromboembolism. Therefore, a comparison of imaging modalities utilised in theranostic nanoparticles for the different types of thrombosis, and several promising techniques not yet utilised, has been provided.

### Clinically utilised imaging techniques

As previously identified, imaging techniques currently used to diagnose thrombosis include ultrasound (US), computed tomography (CT) and magnetic resonance imaging (MRI) 44. These techniques can identify thromboembolisms with high specificity but can be limited by the location of the thrombus, as well as other factors. For instance, US is especially valuable in the detection of thrombosis, due to its widespread availability, lack of dependence on contrast agents, and non-invasive nature 9. Diagnosis of thrombosis with US can be performed utilising two different methods. Compression US relies on the compression of the vein to identify blood clots, but it is hampered if overlying anatomical structures are present, resulting in a low specificity for thrombosis other than superficial DVT in lower extremities 45. Doppler US on the other hand may be used in a variety of cases, as it can detect changes to blood flow caused by occluding thrombi. However, in both approaches specificity of diagnosis depends heavily on the skills of the operator, as operation and correct identification of thrombi can be complicated. Regardless, several theranostics with US-enhancing capabilities have been developed, as they can also relatively easily be produced by the use of microbubbles, as previously explained 32, 34, 38, 40, 41.

Unfortunately, some of these systems suffer from one of the main concerns affecting theranostics: diagnostic/therapeutic dose discrepancy. For instance, Zhong *et al.* administered rats with a dose of 4 mg/kg of their US-observable nanoparticle to exert their thrombolytic efficacy 34. In comparison, clinically used US contrast agent DEFINITY^®^, which has a similar design, has a recommended dosage of 0.011 mg/kg 46. Though these systems likely display somewhat different characteristics, it is expected that such a relatively high dosage may hinder US imaging of this system due to strong acoustic attenuation. Simultaneously, if the dosage is lowered to enable clinical US imaging, their thrombolytic efficacy may be significantly reduced. Hence, theranostics must be designed to use an imaging modality and therapeutic which can both be loaded in their required dosage, as to avoid this diagnostic/therapeutic dose discrepancy.

Besides US, CT has historically also seen a lot of use in the diagnosis of a wide range of thrombi, though use in theranostic systems has been limited to a single publication. Due to its ability to quickly obtain high-quality images with a high depth of penetration, CT is frequently used for severe acute embolisms requiring rapid diagnosis. Therefore, Chang *et al.* developed a gold nanorod (AuNR) theranostic system which displayed CT contrast capabilities alongside antithrombotic efficacy 47. However, the use of this system, and the use of CT for diagnosis of thrombosis in general, suffers from some major limitations. CT relies on potentially harmful ionising radiation, thus it cannot be used frequently for a patient. More prominently, is the lack of sensitivity of CT, which means a large dosage of contrast agent is required. This once again leads to diagnostic/therapeutic dose discrepancies, though reversed compared to US imaging. Chang *et al.* showed this, where their theranostic system required concentrations far above the therapeutically relevant dosage to be visible in CT imaging 47. This may cause toxicity issues, and can result in prohibitive costs associated with such a large dosage, indicating why few theranostic systems are designed for use with CT.

MRI on the other hand has been used fairly extensively as an imaging modality for theranostic nanoparticles for thrombosis management. Compared to CT, MRI allays the need for ionising radiation and provides more structural information 45. The presence of a thrombus in MRI can again be determined through disruptions in blood flow, though direct imaging of thrombi has also been proven possible 45. MRI contrast agent-containing thrombosis theranostic nanoparticles have been researched extensively, focusing primarily on the use of IONPs as previously mentioned 31-33, 35. Unfortunately, MRI also has limitations in its use. MRI has many contraindications, reducing the number of patients that can be scanned with this technique 48. Additionally, MRI has a much longer imaging time than CT, thus it is less suited for emergency situations, where rapid diagnosis is key to preventing mortality and morbidity. Hence, MRI-observable theranostic systems designed for prevention of thrombosis, such as the system developed by Groult *et al.* 31, have more clinical relevance than those aimed at the treatment of acute thromboembolisms 33, 34, where the time to diagnosis is critical. Finally, MRI suffers from a lack of availability due to prohibitive costs associated with the equipment. Therefore, it is generally reserved for cases where US or CT cannot be applied 49.

Thus, all imaging techniques which see clinical use for the diagnosis of thrombosis exhibit limitations, as outlined in **Table 2**. Therefore, no one technique can be used to diagnose all types of thrombosis. However, if a theranostic agent is designed to treat a specific type of thrombosis, these conventional imaging techniques may still be of use if chosen correctly. Regardless, it may also be beneficial to consider utilising newer, emerging imaging techniques when designing theranostic nanoparticles for thrombosis, as these can overcome some of the limitations posed by the conventional approaches.

### Emerging imaging techniques

Several other techniques besides MRI, CT, and US have been utilised in theranostic nanoparticles for thrombosis. It is important to note that these techniques are not yet clinically applied for the diagnosis of thrombosis, thus their specificity is yet to be determined. These systems most prominently include optical imaging and photoacoustic imaging (PAI). Optical imaging allows for high sensitivity and spatial resolution but is practically limited to *ex vivo* applications due to its low depth of penetration 50. PAI seeks to resolve this by combining the spatial resolution of optical imaging with the depth of penetration of US. When tissue containing a photo-absorber is exposed to light of a specific wavelength, the absorption of light causes the tissue to heat up and thus slightly expand, followed by rapid cooling and contraction to the original state. This expansion and contraction cause sound waves, which can be measured at a depth of up to 7 cm 51. PAI enables clinically relevant imaging at resolutions several times higher than CT or MRI 52 and can be used to diagnose thrombosis in upper extremities, where US is limited due to the presence of overlying anatomical structures preventing compression of veins 45.

**Figure 4:**
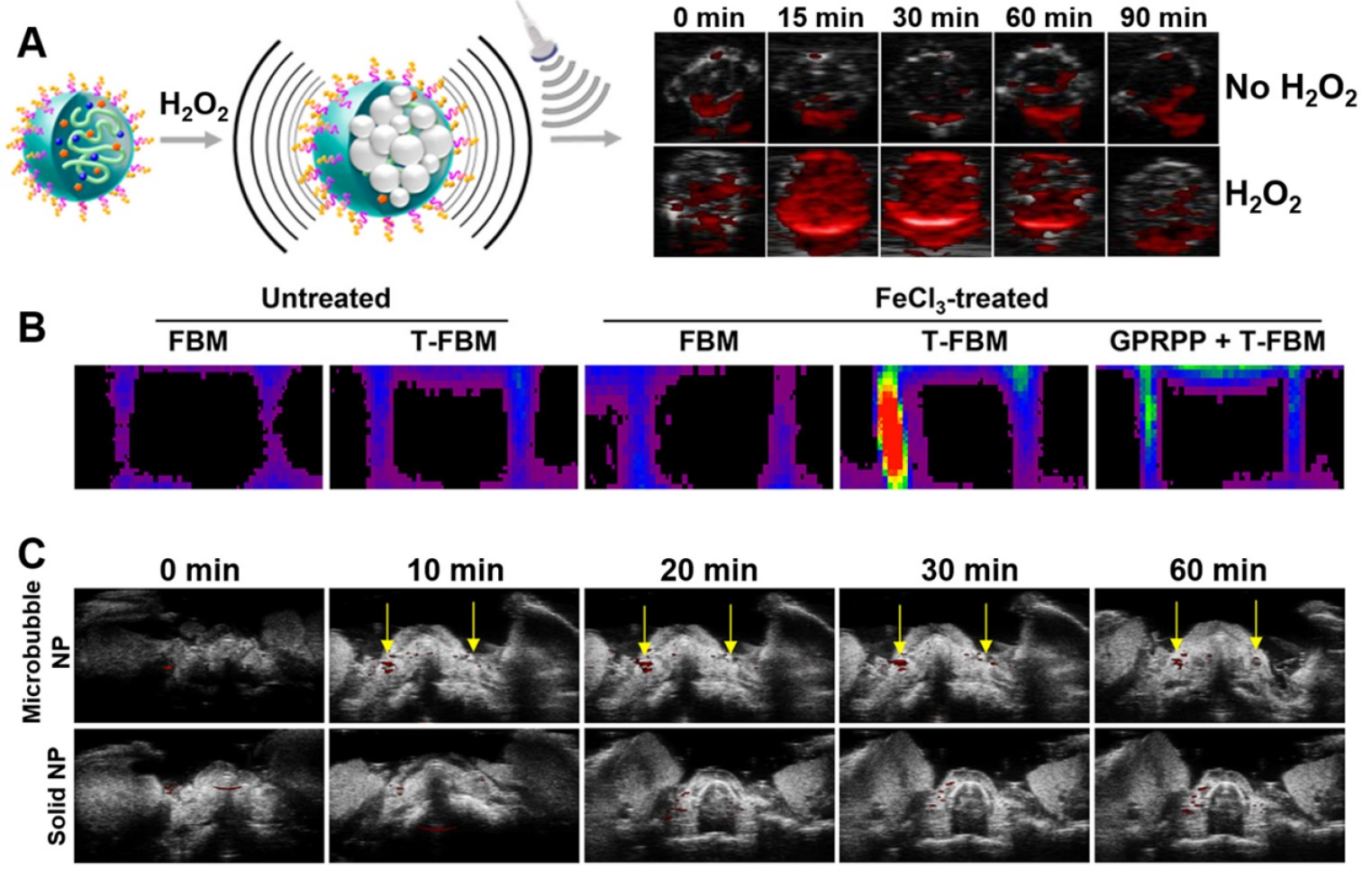
** H_2_O_2_-responsive, PAI contrast enhancing theranostic nanoparticle system (FBM) incorporating fibrin-targeting GPRPP peptides. A.** Schematic representation of activation of the system in the presence of H_2_O_2_, and subsequent amplification of PAI signals, with PAI images indicating H_2_O_2_ responsive imaging. **B.** Fluorescence imaging of carotid artery with thrombi (“FeCl_3_-treated”) and control (“untreated”) after injection of non-targeted nanoparticles (FBM), targeted nanoparticles (T-FBM), and targeted nanoparticles with excess targeting peptide saturating targeted receptors. **C.** Time course of PAI imaging of carotid arterial thrombosis after injection of microbubble or solid nanoparticles loaded with dye, demonstrating the PAI-enhancing effect of microbubbles. Adapted with permission from Jung *et al.* 53, Copyright 2019 American Chemical Society.

PAI facilitates direct imaging of thrombosis due to the presence of natural photo-absorbers in thrombi, however, the signal-to-background ratio and specificity can be further improved through the introduction of exogenous contrast agents. Thus, several theranostic nanoparticles containing PAI contrast agents have been developed for thrombosis 32, 41, 53-55. Most of these systems rely on the conjugation of a fluorescent dye to the particles, enabling high contrast tracking of their antithrombotic activity. Perhaps the most promising systems of all was developed by Jung *et al.* (**Figure 4**) 53. This system showed impressive responsive behaviour to the presence of H_2_O_2_, which is a biomarker of thrombosis (**Figure 4A**). Additionally, it exhibited thrombus targeting in a FeCl_3_-induced carotid arterial thrombosis mouse model (**Figure 4B**). However, perhaps most interesting, is that Jung *et al.* showed that the use of microbubbles could further enhance PAI contrast of a dye *in vivo* compared to a dye conjugated to commonly used solid nanoparticles (**Figure 4C**). However, this system does have increased complexity of fabrication, reducing its cost-effectiveness compared to the other, simpler systems. Therefore, there is a need for further research to provide a system with both good imaging and simplicity of production, as this may result in a clinically relevant PAI observable theranostic system.

Despite the numerous advantages of PAI, it will likely be restricted to the detection of thrombi in limbs and the carotid artery due to its limited depth of penetration 56. Thus, the application of imaging modalities with a higher depth of penetration is of great interest. One option in this regard is the use of ^19^F MRI, where Myerson *et al.* produced two papers studying theranostic nanoparticles for thrombosis prevention with ^19^F MRI capabilities 57, 58. Where conventional MRI lacks sensitivity, ^19^F MRI compensates for this with a low endogenous background signal, enabling easier detection of the contrast agent 59. Myerson *et al.* exploited this by producing perfluorocarbon-containing nanoparticles for the delivery of PPACK, a thrombin inhibitor 57, 58. Interestingly, this design is very similar to the nanodroplets designed for US-enhanced cavitation as previously described 40, 41, though here perfluoro-15-crown-5-ether (PFCE) was used instead of PFP. Hence, such PFCE-containing nanoparticles can also undergo a phase change to produce microbubbles, enhancing US contrast 60. Vice versa, the PFP-containing nanodroplets designed for cavitation may also be observable in ^19^F MRI 61. However, this is unlikely to be clinically relevant, as these cavitating agents are designed for rapid treatment of acute thromboembolisms and would thus be limited by the slow imaging time of ^19^F MRI. In contrast, the systems designed by Myerson *et al.* are intended to halt disease progression in non-acute thrombosis, thus imaging with MRI is still practical despite its longer imaging time 57, 58. Unfortunately, the clinical use of these nanoparticles is somewhat restricted, as ^19^F MRI suffers from a lack of availability even more so than conventional MRI, due to the need for specialised coils and hardware 59. Regardless, the use of ^19^F MRI observable theranostic agents for preventative treatment for thrombosis appears promising due to the lower background noise than ^1^H MRI, while retaining its depth of penetration and favourable safety profile 59.

However, as previously mentioned, all forms of MRI are limited to diagnosis of non-acute thrombosis, due to their long imaging time. Hence, it may be beneficial to consider the use of fast imaging techniques. To this end, the use of imaging modalities which are yet to be applied to theranostics may be of benefit. This includes single-photon emission computed tomography (SPECT), positron emission tomography (PET), and magnetic particle imaging (MPI).

**Figure 5:**
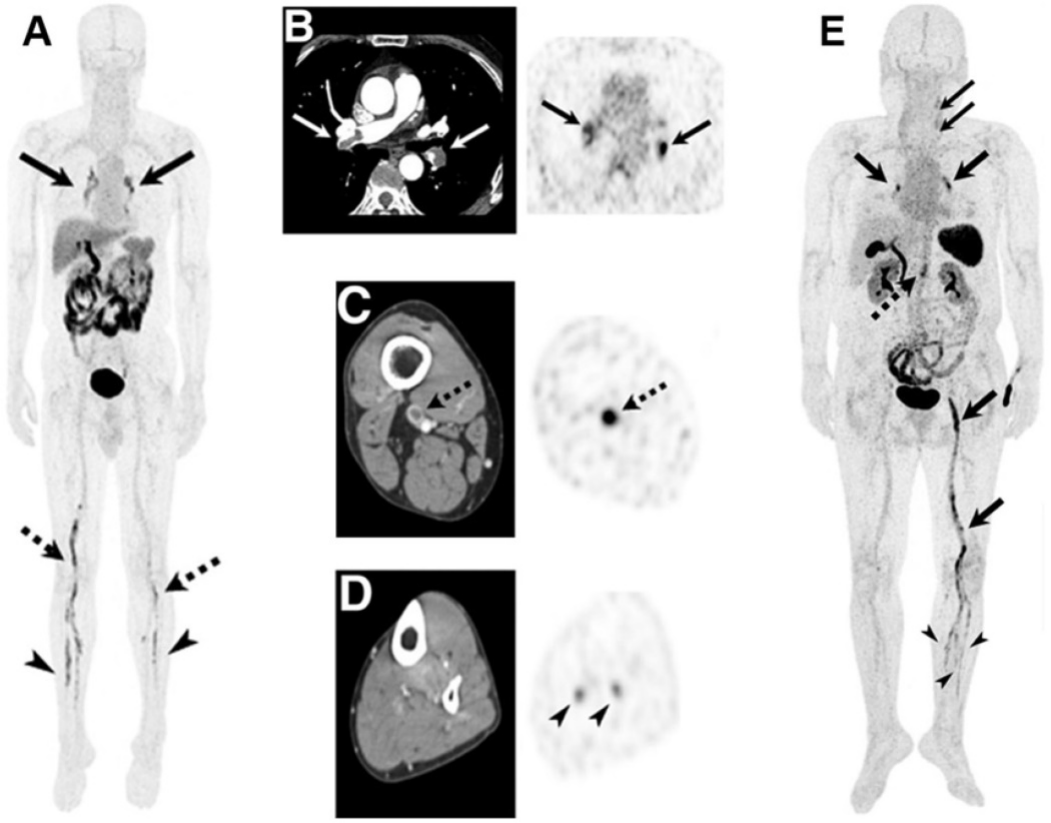
** PET/CT imaging of DVT and pulmonary embolism (PE) using activated glycoprotein IIb/IIIa targeting ^19^F-GP1. A.** PET/CT of a 55-year-old man with DVT and PE. Blood clots were identified through the accumulation of tracer in pulmonary arteries (solid arrows), and in proximal (dotted arrows) and distal (arrowheads) veins of legs. **B-D.** Transaxial CT images (left) clearly show blood clots in pulmonary arteries **B.** and proximal vein **C.** Uptake of tracer determined through PET (right) confirms blood clots identified through CT (**B**, **C**), and identifies thrombi in distal veins not observable in CT **D. E.** PET/CT of a 69-year-old woman identifying thrombi in pulmonary arteries and veins of lower left extremities (DVT) (thick arrows); left common carotid artery (narrow arrows); left anterior tibial, posterior tibial and peroneal veins (arrowheads); and abdominal aorta (dotted arrow). Adapted with permission from Kim *et al.* 66, copyright 2019 SNMMI.

SPECT and PET both function by detecting radiation emitted from radioisotopes, which can be rapidly detected with an unlimited depth of penetration and high sensitivity 50. Comparatively, SPECT boasts more widespread availability and ease-of-handling of isotopes, whereas PET provides superior sensitivity and resolution 62, 63, though recent advances in SPECT technology are closing that gap 64. Unfortunately, both PET and SPECT exhibit some limitations surrounding their dependence on ionising radiation, the requirement of radiolabelling facilities, and a somewhat lacking spatial resolution compared to PAI and MRI 65. Additionally, they cannot obtain structural information, though this can be mitigated by using multimodal SPECT/CT and PET/CT. Such an approach has been applied in a clinical trial of a non-theranostic imaging probe for the diagnosis of thrombosis, where an ^18^F-labelled thrombus-targeting ligand was utilised for PET/CT imaging 66. As apparent in **Figure 5A-C,E**, this approach enabled the facile identification of a range of different thrombi. Additionally, PET/CT could detect blood clots in distal veins normally missed by conventional CT imaging (**Figure 5D**) 66. Hence, it is expected that such an approach could be highly beneficial if applied in theranostics as well.

Another highly promising, emerging imaging technique that circumvents the need for ionising radiation while retaining the unlimited depth of penetration, is magnetic particle imaging (MPI) 67. MPI directly quantifies the presence of magnetic nanoparticles, where the lack of magnetism of native tissues means high-sensitivity images can be taken at any depth. Similar to SPECT and PET, no structural information is obtained, thus image analysis is simplified compared to techniques such as MRI. Scanning times are also drastically reduced while sensitivity is increased compared to MRI, reducing the time to diagnosis and thus the chance of mortality and morbidity 67. MPI is yet to see widespread clinical use, which explains why no theranostic nanoparticles have been tested for MPI diagnosis of thrombosis. However, multiple imaging probes for thrombosis have already been developed based on IONPs 68, 69, and the development of several theranostic systems for other diseases utilising MPI has produced promising results 70, 71. Therefore, it is expected that the implementation of such techniques for the development of theranostic nanoparticles for thrombosis is also possible and may be highly beneficial.

An alternative approach of high value to the accurate diagnosis of thrombosis is the use of multimodal imaging. Here, multiple imaging techniques can be utilised simultaneously, compensating for each other's limitations and offering synergistic advantages. Unfortunately, multimodal imaging is complicated by the need for the coadministration of contrast agents with different pharmacokinetic profiles 72. Therefore, the use of a single imaging probe with multimodal imaging capabilities is preferred 72, and theranostic nanoparticles are ideal candidates in this context. Hence, multimodal theranostic nanoparticles have been developed for thrombosis, using optical/PAI 41, 54 and MRI/PAI/US 32, 34.

Unfortunately, introducing multiple imaging moieties into one nanoparticle system increases the complexity of fabrication, reducing the cost-effectiveness of these multimodal theranostic nanoparticles 73. Additionally, as the sensitivities of the imaging techniques vary, loading of each imaging moiety must be optimised individually to provide adequate signals in both imaging techniques 74. Inadequate optimisation leads to discrepancies in dose requirements for each imaging technique, as experienced by Bai *et al.* and their theranostic probe. 41. Thus, research into multimodal theranostic nanoparticles for thrombosis is highly promising but requires further development to reach its clinical potential.

Overall, the diagnosis of thrombosis is an expanding field with exciting developments, many of which are being driven by the use of theranostic nanoparticles. Unfortunately, none of the imaging techniques employed for theranostic nanoparticles is without limitations, where alternative imaging techniques may provide improvements but again come with drawbacks in safety and availability. Hence, there is not one superior imaging technique capable of diagnosing all types of thrombi in a favourable manner. Therefore, the main consideration when choosing an imaging modality for theranostic nanoparticles should be what type of thrombosis it is aimed to diagnose and treat. Particularly, the location and acuity of thromboembolism are of importance, where the most suitable imaging technique should be chosen accordingly. Finally, the loaded dosage of the imaging moiety and therapeutic must be considered to avoid diagnostic/therapeutic dose discrepancy, which is a major hurdle in the clinical translation of theranostic systems.

## Responsive theranostic nanocarriers

Another approach towards improving theranostic nanoparticles for thrombosis is the use of internal or external stimuli-responsive nanocarriers. By enriching theranostic systems with stimuli-responsive behaviour, the therapeutic effect of the system may be turned on as required, decreasing side effects without negatively impacting efficacy. Hence, several theranostic nanocarriers with responsive behaviour have been designed, with promising results. Four stimuli have been utilised for this purpose to date, namely US, H_2_O_2_, thrombin, and light-induced hyperthermia, each with its advantages and disadvantages. Hence, these systems are compared here, with a focus on their specificity, ease of use, and ease of incorporation, as these directly affect the safety, applicability, and cost-effectiveness of the systems. Furthermore, a short overview of alternative stimuli which have seen use in drug delivery systems for thrombosis, but are yet to be applied to theranostics, is also provided.

### Ultrasound (US) as a stimulus

As apparent from **Table 1**, most stimulus-responsive theranostic nanoparticles rely on stimulation by low-intensity focused ultrasound (LIFU), and for good reason. As an external stimulus, LIFU provides excellent specificity due to a lack of endogenous stimulation. Furthermore, LIFU is also known to improve thrombolysis by PFC-containing nanodroplets through enhancement of the cavitation effect, thus exerting a therapeutic effect. Upon stimulation with LIFU, the PFC in these nanodroplets expands, causing them to explode and rapidly cause thrombolysis. Unfortunately, there is also potential for tissue damage upon cavitation, thus Wang *et al.* developed a theranostic with US-responsive release of a thrombolytic without involving cavitation 39. This system relies on US-induced stable oscillation of the microbubble, which results in a highly specific and extended-release of tPA, as opposed to the burst effect of PFC-containing nanodroplets 39.

The release profile depends on the application of the theranostic, where, for instance, systems for preventative treatment benefit from an extended-release profile, increasing the period of protection against thrombosis. In contrast, treatment of acute thromboembolisms through the delivery of thrombolytics should follow a more rapid release to degrade the thrombus as fast as possible. Nonetheless, burst release may not be ideal, as rapid degradation may lead to fragmentation of the thrombus, followed by occlusion of smaller vessels 75. Thus, the release profile of the responsive theranostic system should be considered to maximise the required efficacy and reduce the side effects of the system.

Unfortunately, it is not possible to directly compare the thrombolytic efficacy of these LIFU responsive systems, as different *in vivo* models were used, though they all appeared quite effective 32, 34, 39-41. Hence, no comment can be made on which system is preferred based on solely their efficacy. However, the system developed by Wang *et al.* may be the most promising, as it does not rely on the potentially dangerous cavitation effect to exert its thrombolytic potential, hence increasing its safety profile 39. Furthermore, this system relies on the same US used for diagnostic imaging, whereas the other systems rely on LIFU. Application of LIFU requires different apparatus, and cannot be performed during US imaging, hence stimulation must be seized before imaging. Thus, the system proposed by Wang *et al.* is likely more clinically applicable than LIFU-responsive systems. Unfortunately, the application of all these systems is somewhat limited by the need for trained practitioners to apply the stimulus. Systems responsive to internal stimuli, in contrast, require little to no effort for responsive release to occur, as pathophysiological markers are utilised as stimuli instead. Thus, two biomarkers of thrombi have been utilised as stimuli of responsive theranostic nanoparticles for thrombosis.

### H_2_O_2_ as a stimulus

Due to extensive efforts from the Lee group, H_2_O_2_ is the most utilised thrombosis biomarker for stimulation of drug release in theranostic nanoparticles, and these particles have shown great promise for preventative treatment for thrombotic events 53, 54, 76. Thrombus formation elevates oxidative stress through a burst release of reactive oxygen species (ROS) which are known to promote platelet activation 77, 78. Therefore, ROS are upregulated in the vicinity of thrombi, and H_2_O_2_ is the most stable ROS and thus the most abundantly present, making it an excellent stimulant for thrombus-responsive drug release 54.

All H_2_O_2_-responsive theranostic nanoparticles for thrombosis have taken the approach of exploiting polymers containing H_2_O_2_-cleavable functional groups. These systems exhibit extended-release of antithrombotics - where the release rate depended on the stability of the employed H_2_O_2_-cleavable group. For instance, Lee *et al.* utilised a polymeric system containing peroxylate esters, which resulted in extended-release over several days 76. In contrast, two theranostic systems utilising boronate esters saw release over several hours instead 53, 54. Hence, the system designed by Lee *et al.* and the use of peroxylate esters in general may have more clinical potential, as it provides extended protection against thrombosis compared to boronate ester-based systems. Besides just causing release, all these systems are also capable of H_2_O_2_ scavenging as H_2_O_2_ is consumed upon cleavage of said functional groups. This reduces H_2_O_2_ levels and thus exerts an antithrombotic effect in addition to the released payload, where again a longer cleavage profile would be preferred to extend the period of protection 53, 54, 76. Regardless, all systems showed impressive antithrombotic efficacy in FeCl_3_-induced carotid arterial thrombosis mouse models.

Though results from these studies appear promising, H_2_O_2_-responsive nanoparticle systems suffer from one inherent disadvantage: a lack of specificity to the pathophysiology of thrombosis. ROS, including H_2_O_2_, as inflammatory markers are involved in a large variety of diseases, including cancer 79, neurodegenerative 80, and autoimmune diseases 81. Hence, specificity of thrombus-responsive release from H_2_O_2_-responsive theranostics may be drastically reduced in patients also suffering from such diseases, leading to an increase in side effects in an already vulnerable population. Additionally, low levels of H_2_O_2_ function as signalling molecules that are involved with the regulation of normal physiology 82, 83. Therefore, if H_2_O_2_ scavenging causes H_2_O_2_ levels to drop below these normal levels other side effects may occur, though further research is required to confirm this. Regardless, the use of a stimulus with higher specificity to thrombosis would be preferable to improve the safety of the responsive theranostic system.

### Thrombin as a stimulus

One particularly interesting internal stimulus for this purpose is thrombin. As previously mentioned, thrombin facilitates the cleavage of fibrinogen to fibrin as part of the coagulation cascade and is also involved in the platelet cascade. Hence, thrombin presence is upregulated when a thrombus is present 2. Subsequently, this increased presence of thrombin may be exploited to introduce thrombus-responsiveness into nanoparticles by incorporating a thrombin-cleavable peptide 84. This peptide mimics thrombin's natural binding site on fibrinogen, which contains a proline-arginine-glycine (PRG) motif 84-88. Such an approach can be highly beneficial as thrombin is specific to thrombosis pathophysiology 89, and the use of enzymes as stimuli is known to result in high specificity and efficiency 90.

**Figure 6:**
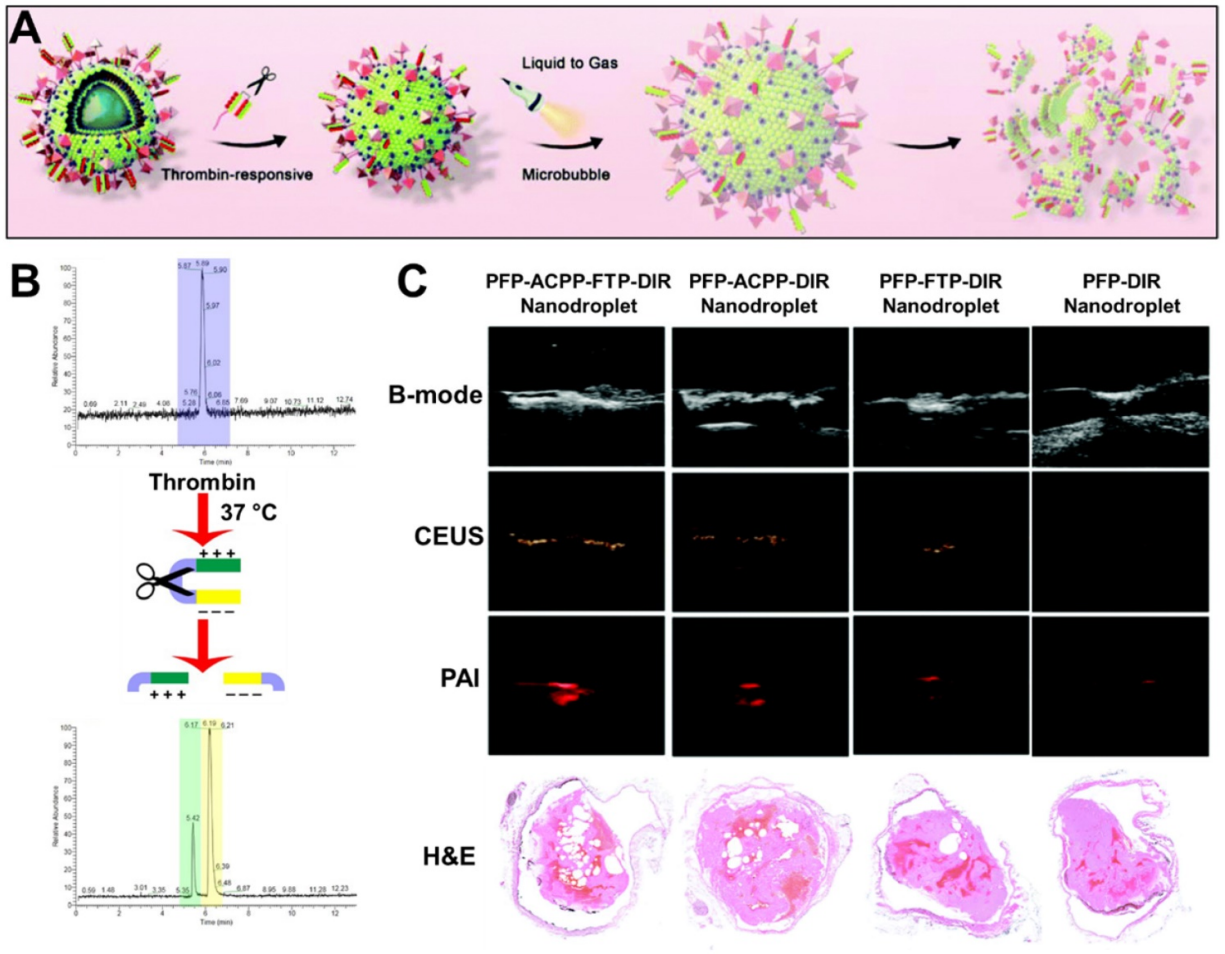
** Theranostic nanodroplets with thrombin-responsive penetration. A.** Schematic overview of nanodroplet with thrombin-responsive penetration, LIFU-responsive phase changing, and subsequent bursting. **B.** Cleavage of the activatable cell-penetrating peptide (ACPP, blue) by thrombin to separate the cell-penetrating peptide (green) from the anionic inhibitory domain (yellow), as verified by liquid chromatography-mass spectrometry. **C.** Imaging of thrombi after treatment with dye-labelled nanodroplets (PFP-DIR Nanodroplet) with ACPP and/or fibrin targeting peptide (FTP). Images were obtained 5 minutes after administration in an inferior vena cava thrombosis rat model. *In vivo* imaging included B-mode US, contrast-enhanced US (CEUS), and PAI. *Ex vivo* imaging included H&E staining of inferior vena cava thrombosis. Adapted with permission from Yang *et al.* 40, Copyright 2020 Royal Society of Chemistry.

Thus, such an approach has been applied by Yang *et al.* to produce a thrombin-responsive theranostic nanoparticle 40. This system is based on a PFP-containing nanodroplet, decorated with activatable cell-penetrating peptides (activatable CPP) which are masked with an inhibitory domain connected through a thrombin-sensitive peptide (**Figure 6A**). Upon cleavage of this peptide by thrombin, the inhibitory domain is released, resulting in exposure of CPP (**Figure 6B**). CPP is known to enhance cellular penetration in a wide variety of tissues, thus this targeting approach can also cause dangerous side effects 40. However, in this system, the presence of the inhibitory domain prevents such non-specific penetration, where only in the presence of thrombin the peptide is cleaved, exposing the CPP. Therefore, cellular penetration is only enhanced at the site of thrombosis, effectively creating a highly specific thrombin-responsive targeting system with enhanced penetration into the dense thrombus. The US and PAI contrast abilities of this PFP nanodroplet theranostic enabled effective monitoring of the distribution of these particles. Hence, its accumulation at the site of thrombosis allowed for high contrast imaging of thrombi in an inferior vena cava thrombosis rat model (**Figure 6C**). A dual-targeting approach with a fibrin-targeting peptide (FTP) further enhanced site-specific accumulation, though it had less effect on penetration into the thrombus 40. Ultimately, the responsive and dual active targeting of these theranostic nanoparticles enabled enhanced breakdown of the clot, as apparent from *ex vivo* imaging of the blood clot.

Besides this approach towards thrombin-responsive targeting, thrombin-responsive drug release has not yet been utilised in theranostic nanoparticles. Several non-diagnostic drug delivery systems have incorporated such functionality though, with promising results indicating this may also be beneficial if used in theranostic nanoparticles. For instance, Xu *et al.* developed a system where recombinant tPA (rtPA) was attached to the surface of the nanoparticles through a thrombin cleavable peptide 88. Hence, the addition of thrombin resulted in a highly specific and rapid release profile. Again, the safety concerns relating to such a fast release should be considered, as fragmentation of the thrombus may occur 75. On the other hand, even the highest concentration of thrombin resulted in 80% release over 2 hours, thus it is expected that release is not fast enough to result in such unwanted events. It did still enable highly efficient thrombolysis, as tested in a FeCl_3_-induced carotid arterial thrombosis mouse model.

Perhaps the most interesting part of this system is that the thrombin-induced peptide cleavage resulted in the exposure of CPP, similar to the theranostic nanoparticles developed by Yang *et al.* 40. Thus, penetration was again greatly increased compared to the free thrombolytic. The combination of this thrombus penetration with the thrombin-responsive drug release means the system developed by Xu *et al.* is more sophisticated and likely more efficacious than the theranostic nanoparticle developed by Yang *et al.* However, with this increased sophistication also comes an increase in the difficulty of production, likely making them less cost-effective. Additionally, the theranostic nanodroplet have the advantage of inherent US-contrasting abilities, where the approach taken by Xu *et al.* would require additional functionality to enable tracing through imaging, further decreasing its feasibility as a theranostic. Finally, this drug delivery system relies on surface loading, likely increasing side effects and degradation of the payload as compared to the theranostic nanodroplet 88. Therefore, further research is required to produce a simple, core-loaded thrombin-responsive theranostic. It is expected that such a system may have high clinical potential, due to the specificity and ease-of-use of thrombin-responsive nanoparticles.

### Light-induced hyperthermia

Hyperthermia as a trigger for responsive behaviour has also been investigated extensively and is of particular interest as the increase in temperature also enhances thrombolysis, aiding in the breakdown of the clot. The simplest systems rely on the direct application of heat, though such an approach can result in low specificity, as the difference between physiological and stimulated temperature is kept small to avoid damage to tissue 91, 92. Alternatively, stimulating heating of the nanoparticle itself, rather than its environment, has also been investigated to increase specificity. Chang *et al.* applied this concept in their theranostic nanoparticles, where urokinase containing gold nanorods (AuNRs) could be heated upon stimulation with near-infrared (NIR) light 47. This facilitated enhanced clot lysis by stimulating localised hyperthermia 47. However, this theranostic system did not contain any heat-responsive materials, thus the urokinase release was non-specific and relatively unresponsive.

Fang *et al.* showed with their non-theranostic drug delivery system that it is possible however 93. Here, a light-responsive hair-derived nanoparticle was loaded with urokinase, and encapsulated in gelatin, which melted and released the thrombolytic payload upon heating. This resulted in particles with a highly specific release profile, and their *in vivo* thrombolytic ability could be controlled through NIR radiation 93. Hence, the adaptation of such a system to also exhibit imaging functionality may result in a highly promising theranostic approach. However, it should be noted that the FeCl_3_-induced femoral vein thrombosis mouse model utilised involved exposing the vein, thus NIR light could be applied directly to the blood vessel. This is unrealistic in a clinical setting, and as tissue penetration of NIR light is limited to <1 cm 94, this drastically limits the applicability of such a system. Therefore, induction of hyperthermia with an alternative stimulus with a higher depth of penetration is desirable.

### Alternative stimuli

As identified, all four stimuli so far applied in responsive theranostic nanoparticles for thrombosis suffer from disadvantages, mostly related to safety concerns, complexity of use, and limited applicability limiting their clinical potential. Hence, the use of alternative stimuli may be of interest to increase their translational potential. Several other stimuli have been used in drug delivery systems for thrombosis without seeing translation into theranostic nanoparticles. Such approaches may be worth considering for application in theranostics as well, as they have shown great promise to reduce the side effects and improve the efficacy of these drug delivery vehicles.

#### Magnetic field stimulus

As opposed to light, a magnetic field does not suffer from a low depth of penetration and can be employed as an alternative external stimulus for hyperthermia. This approach relies on alternating magnetic fields to stimulate the heating of magnetically responsive materials, such as IONPs. Due to the unlimited depth of penetration, ease of manipulation of magnetic fields, and lack of magnetism of tissue, localised heating can be performed highly specifically at virtually any position in the body without damage to healthy tissue. Hence, such an approach has even reached clinical success in other diseases, with phase III clinical trials testing magnetically-induced hyperthermia in nanoparticles for the treatment of prostate cancer 95. The technique requires information about the location of the thrombolytic event before treatment can commence, however, as IONPs also function as an MRI or MPI contrast agent 96, 97, this is a great example of the merit of IONPs as a theranostic agent 98. Additionally, targeting of the IONPs to thrombi using manipulation of the magnetic field may also be possible, though caution should be taken to avoid particle aggregation and subsequent vessel occlusion as will be further explored later 91, 99. Unfortunately, the equipment required to perform such magnetic field manipulation is not yet widely available, though this will likely increase over time. Hence, it is expected that the application of magnetically-induced hyperthermia responsive nanoparticles to create thrombosis theranostics may lead to exciting opportunities, where highly sophisticated yet simple systems may be developed.

#### Secreted phospholipase A_2_ as a stimulus

Besides thrombin, several other enzymes are upregulated in the thrombogenic environment. This includes secreted phospholipase A_2_ (sPLA_2_), which is produced from activated platelets and inflammatory cells 100. sPLA_2_ cleaves the ester bonds in glycerophospholipids, hence a liposome based on such glycerophospholipids showed sensitivity to sPLA_2_, and could be used to produce an sPLA_2_ responsive drug delivery system 101. Such a system is far easier to produce than a thrombin responsive system, which requires the synthesis and incorporation of a thrombin-labile peptide. Additionally, the payload is loaded in the core of these particles rather than on the surface, reducing degradation and exposure to non-target tissue. However, as liposomes are prone to leakage of the payload, this system does show non-specific release 101. Additionally, sPLA_2_ is not specific to the thrombogenic state, as it is also present in stable atherosclerotic plaques 100. Hence, although such an sPLA_2_ responsive system is easier to produce than the thrombin systems discussed, it could also suffer from a lack of specificity, potentially causing safety concerns.

#### Low pH as a stimulus

Upon occlusion of a blood vessel by a thrombus, the blood surrounding the thrombus becomes oxygen-starved, resulting in a lowered pH 102. Several drug delivery systems have exploited this reduction in pH as a stimulus for drug release, by covalently conjugating the drug to the nanoparticle through a pH-sensitive imine 103, 104. The immobilised drug has a reduced activity, which is restored upon cleavage of the imine. However, the release of the drug is not highly specific, as this cleavage also occurs at physiological pH, albeit at a slower rate 103. Additionally, such an acidic environment is not specific to thrombosis, where, most notably, tumour microenvironments also exhibit a lower pH 105. Hence, such a system may have limited applicability in patients suffering from cancer. Altogether, this approach may be easily incorporated, but the investigation into more specific responsive systems is likely to be more fruitful.

#### Shear as a stimulus

Besides causing an increase in the beforementioned biomarkers, thromboembolisms also cause an altered mechanical environment. Upon (partial) occlusion of a blood vessel by the thrombus, localised shear rate increases as a result, which has been used as a stimulus to disrupt nanoparticles, resulting in the release of therapeutics. Such an approach was first used by Korin *et al.*, where a microaggregate of nanoparticles could be disrupted by shear to enable adhesion to vessel walls and subsequent accumulation of the nanoparticles at the site of embolism 106. A recent application of a shear responsive system further improved this approach, where Zhang *et al.* developed a dual-payload drug delivery system displaying highly specific shear-responsive release of a thrombolytic, and extended release of an antiplatelet agent 107. This enabled rapid treatment of acute thromboembolisms, while providing prolonged protection against recurrent thrombogenesis. Additionally, due to the specificity of release, this system significantly reduced the haemorrhagic risk compared to the free thrombolytic (UK) and antiplatelet (TI) in a tail bleeding assay 107. Hence, such an approach may be highly promising for the production of theranostic nanoparticles with a good safety profile. Unfortunately, an increase in blood shear stress is often also a result of other CVDs, such as hypertension and aneurysms 108, 109, thus the applicability of shear responsive systems may be limited in patients suffering such diseases. The double-shell design employed here also increases the complexity of fabrication and thus reduces the cost-effectiveness, though the development of a more simplistic design may negate this issue.

The introduction of stimuli-responsiveness is an exciting prospect for the treatment of thrombosis with high efficacy and no side effects. Several responsive approaches have been investigated for thrombosis, either using theranostic or non-theranostic nanocarriers. Particularly, the use of thrombin and magnetically-induced hyperthermia have shown great promise to enable specific release. However, further research is needed to further improve the translational potential of responsive approaches. Current limitations lie mostly in the safety, simplicity, and cost-effectiveness of such responsive systems, due to a lack of specificity, the need for external input from a trained professional, and complex fabrication. Therefore, further research is vital to ensure future clinical translation of such responsive systems. Specifically, the investigation into stimuli that specifically and solely occur during thromboembolisms, and responsive materials with specific and tunable responses to said stimuli is highly recommended.

## Opportunities for targeted theranostic systems

As apparent from Table 1, many theranostic nanoparticles for thrombosis utilise active targeting strategies. Such strategies involve the attachment of targeting moieties, such as antibodies or peptides, which can target structures of the targeted tissue (thrombi) with high affinity and specificity, selectively increasing the efficiency of delivery to the thrombus without increasing delivery to non-target tissues. Many different targeting moieties have been utilised for this purpose, where fibrin and platelets are common targets due to their prevalence in thrombi. However, one should consider the type of thrombosis the system is aimed to treat when designing a targeted theranostic, as targets vary between thrombus types.

Fibrin is a particularly interesting target due to its prevalence in all types of thrombi 7. Additionally, in normal physiology, fibrin is mainly present in its inactivated form (fibrinogen), which is cleaved by thrombin during the coagulation cascade to become fibrin 2. Thus, if fibrin can be targeted specifically without targeting fibrinogen, selective targeting of a wide range of thrombi can be achieved. To this end, several different targeting moieties have been utilised in theranostic nanoparticles. These mostly focus on the use of peptides, including CREKA 34, 54 and GPRPP 53, 76. Both of these pentapeptides can selectively target fibrin with high affinity, where CREKA enabled *in vivo* accumulation at the site of thrombus as fast as 3 minutes, with maximum accumulation after 30 minutes 54. GPRPP enabled similar results, taking 5 minutes and 30 minutes, respectively, though this difference is likely just due to the measurement intervals 53. Due to these fast accumulation times, systems utilising such ligands for targeting may be useful in emergency use, reducing the time to effective treatment. Additionally, these ligands are also practical, as they are easy to produce and unlikely to stimulate an immune response due to their small size 110.

However, an even faster time to reach maximum accumulation would be preferable, as this would enable a more rapid diagnosis of thromboembolism, where the high concentration of theranostic at the site of thrombus enables easier identification. To this end, the use of larger targeting moieties may be of interest, as these generally exhibit higher targeting affinity. For instance, a fibrin-targeting antibody with promising characteristics has recently been developed 111, the adoption of which may enable the production of fibrin-targeted theranostic agents with a faster time to maximum accumulation. However, antibodies are also associated with some limitations, mostly relating to their inhibitive cost of development and tendency to cause immune responses. Hence, alternative targeting moieties may be worth considering.

An area where antibodies and their fragments have seen adoption is platelet-targeted theranostic nanoparticles. As previously described, platelets play a large role in the formation of thrombi, particularly arterial thrombi. Said platelets contain glycoprotein IIb/IIIa on their surface, which becomes activated upon initiation of thrombogenesis 2. Thus, if activated glycoprotein IIb/IIIa can be selectively targeted, thrombi-homing theranostic nanoparticles can be produced. To this end, a single-chain antibody, scFv_SCE5_, was developed 112. Single-chain antibodies are of relatively smaller size compared to antibodies, and lack the Fc region, drastically reducing their immunogenicity 38, 113. Wang *et al.* utilised scFv_SCE5_ to target a theranostic nanoparticle system specifically to activated glycoprotein IIb/IIIa, thus enabling thrombi-specific targeting 38. This resulted in improved thrombolytic efficacy in a FeCl_3_-induced carotid arterial thrombosis mouse model, while decreasing haemorrhagic risk compared to the free thrombolytic 38. Though this study did not investigate the speed of accumulation, another study reported rapid accumulation in less than 5 minutes, reaching the maximum accumulation in only 10 minutes 114. This is significantly faster than the CREKA and GPRPP fibrin-targeted theranostic nanoparticles. Unfortunately, single chain antibodies are harder to produce than small peptides, thus somewhat reducing their cost-effectiveness comparatively, though this may be worth it for the increased affinity and specificity.

The targeting of platelets with readily available peptides has also been explored. Particularly of interest is cRGD, a short, cyclic peptide that is widely available due to its widespread use throughout nanomedicine in multiple disease areas, most notably for targeting several tumour types that also have a high expression of integrins 115, 116. Thus, several theranostic nanoparticles for thrombosis have applied it too, as cRGD has shown an acceptable affinity for thrombi 33, 41. However, targeting with cRGD has been reported to take 15 minutes for noticeable accumulation, and 120 minutes for maximum accumulation 117. This indicates their affinity for platelets is drastically lower than scFv_SCE5_, which targets the same receptor. This extended time for accumulation means cRGD targeted theranostics may be unsuitable for emergency use, though use in preventative treatment may still be possible, where the time to treatment is not as critical. Alternatively, a dual ligand approach may enable higher affinity, as shown by Zhang *et al.* 118. Here, the combination of cRGD and EWVDV (another platelet targeting peptide) resulted in a significantly improved affinity for thrombi. However, such an approach does increase the complexity of synthesis compared to a single, higher affinity targeting ligand such as scFv_SCE5_. Additionally, as cRGD has also seen application in a wide variety of diseases, it likely has low specificity for thrombi. This may be prohibitive to its use in patients suffering other diseases including cancer due to potential off-site effects. Hence, targeting ligands with a higher specificity may also be preferable.

**Figure 7:**
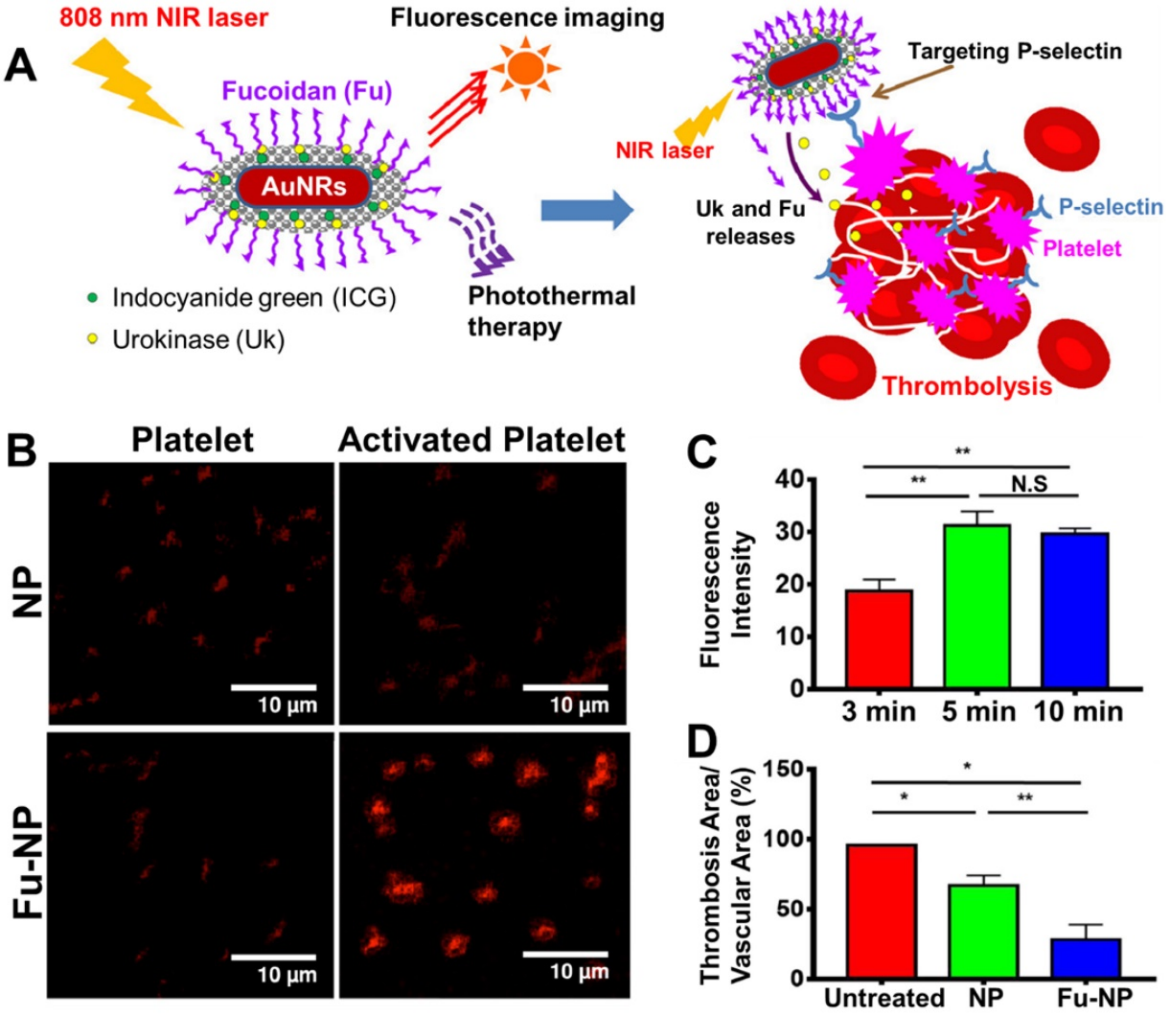
** Fucoidan (Fu) decorated theranostic gold nanorods (AuNRs) for platelet targeted dual delivery of a thrombolytic (urokinase) and an anticoagulant (Fu). A.** Schematic representation of the Fu decorated AuNRs, their delivery to the thrombus, and NIR responsive behaviour. **B.** *In vitro* targeting affinity of non-targeted AuNRs (NP) and Fu targeted AuNRs (Fu-NP) for inactive or activated platelets as determined through confocal imaging. **C.** *In vivo* accumulation of Fu targeted AuNRs overtime at the site of thromboembolism in a ferric chloride-induced mesenteric thrombosis mouse model. Time is measured from the time of injection of the theranostic. **D.** *In vivo* thrombolytic capacity of the non-targeted AuNRs (NP) and Fu targeted AuNRs (Fu-NP) in a ferric chloride-induced mesenteric thrombosis mouse model, as determined by comparing the thrombus area before and after treatment. All data is shown as mean ± SD (n ≥ 3). *p < 0.05, **p < 0.01. Adapted with permission from Chang *et al.* 47, Copyright 2021 Elsevier.

Fucoidan is a promising alternative ligand with a high affinity to platelets and has recently been incorporated in a theranostic nanoparticle (**Figure 7A**) 47. Fucoidan is an anticoagulant derived from algae, which has also seen use as a P-selectin targeting ligand in several drug delivery systems 107, 119 and an imaging probe 120. Thus, Chang *et al.* decorated the surface of their gold nanorod (AuNRs)-based theranostics with fucoidan, where it provides functionality both for targeting and preventative therapy 47. This enabled highly specific targeting of activated platelets with low affinity for their inactive state, as P-selectin expression is turned on upon activation of platelets (**Figure 7B**). The maximum accumulation of these particles at the site of thrombus *in vivo* also took only 10 minutes, indicating fucoidan's high affinity for activated platelets (**Figure 7C**) 47. Hence, this system exhibited impressive thrombolytic capabilities when tested in a ferric chloride-induced thrombosis mouse model (**Figure 7D**). However, the synthesis of these nanoparticles appears to be quite complex, thus limiting their clinical potential due to reduced cost-effectiveness. Additionally, their affinity for thrombi is likely also lower than larger targeting ligands, such as scFv_SCE5_. Nonetheless, as fucoidan itself is relatively easy to produce and also exhibits anticoagulant properties, fucoidan-targeted nanoparticles may still have a high potential for clinical translation.

Another approach that has gathered significant interest is the use of endogenous-derived membrane vesicles for targeting thrombi. By cloaking nanoparticles in membranes derived from cells present in thrombi, active targeting of the thrombus can be performed *via* the natural proteins present on the membrane 121. Thus, theranostic nanoparticles have been developed using RBC 43 and platelet-derived membranes 42. The use of platelet membranes is particularly of interest, where the presence of glycoprotein IIb/IIIa on their surface enables active targeting of thrombi through coagulation with platelets and fibrin 88. This targeting is apparent in **Figure 6**, where the membrane vesicles increase accumulation in the thrombus as determined through *ex vivo* imaging (**Figure 6D**), and subsequently increase thrombolysis, restoring blood flow (**Figure 6C**). Such an approach also has the added benefit of an increased half-life due to the self-markers on these membranes reducing macrophage uptake, leading to circulation times of 90-120 days 43. Hence, the use of such biomimetic materials is highly promising for the development of actively targeted theranostic nanoparticles.

One prominent consideration for actively targeting theranostic nanoparticles towards thrombi is the type of thrombosis. For instance, platelet targeted nanoparticles may have limited applicability for the treatment of venous thrombi, due to the low amounts of platelets present in said thrombi 7. Platelet-targeting peptides are likely limited in this application due to their lower affinity for platelets preventing them from targeting the few platelets present in thrombi. Fucoidan and scFv_SCE5_ targeted theranostics on the other hand may avoid this limitation as their higher affinity may still enable targeting of such low amounts of activated platelets. Therefore, the scFv_SCE5_ and fucoidan targeted theranostic system proposed by Wang *et al.* and Chang *et al.* respectively may be more clinically relevant 38. Platelet membrane cloaked theranostic nanoparticles may also be preferable, as the activated glycoprotein IIb/IIIa on their surface enables affinity for both platelets and fibrin. Alternatively, the development of single-chain antibodies that can target fibrin might also be of interest to develop theranostic nanoparticles with high specificity and affinity for more types of thrombi, including platelet-poor venous thrombi.

Alternatively, active guidance of the particle to the site of thromboembolism through external stimulation such as a magnetic field may enable the targeting of all kinds of thrombi. Such an approach also enables high targeting specificity due to the lack of endogenous stimulation. Magnetic fields are particularly interesting for this purpose due to their high penetration and ease of use, where a magnet can simply be placed on the site of thromboembolism.

**Figure 8:**
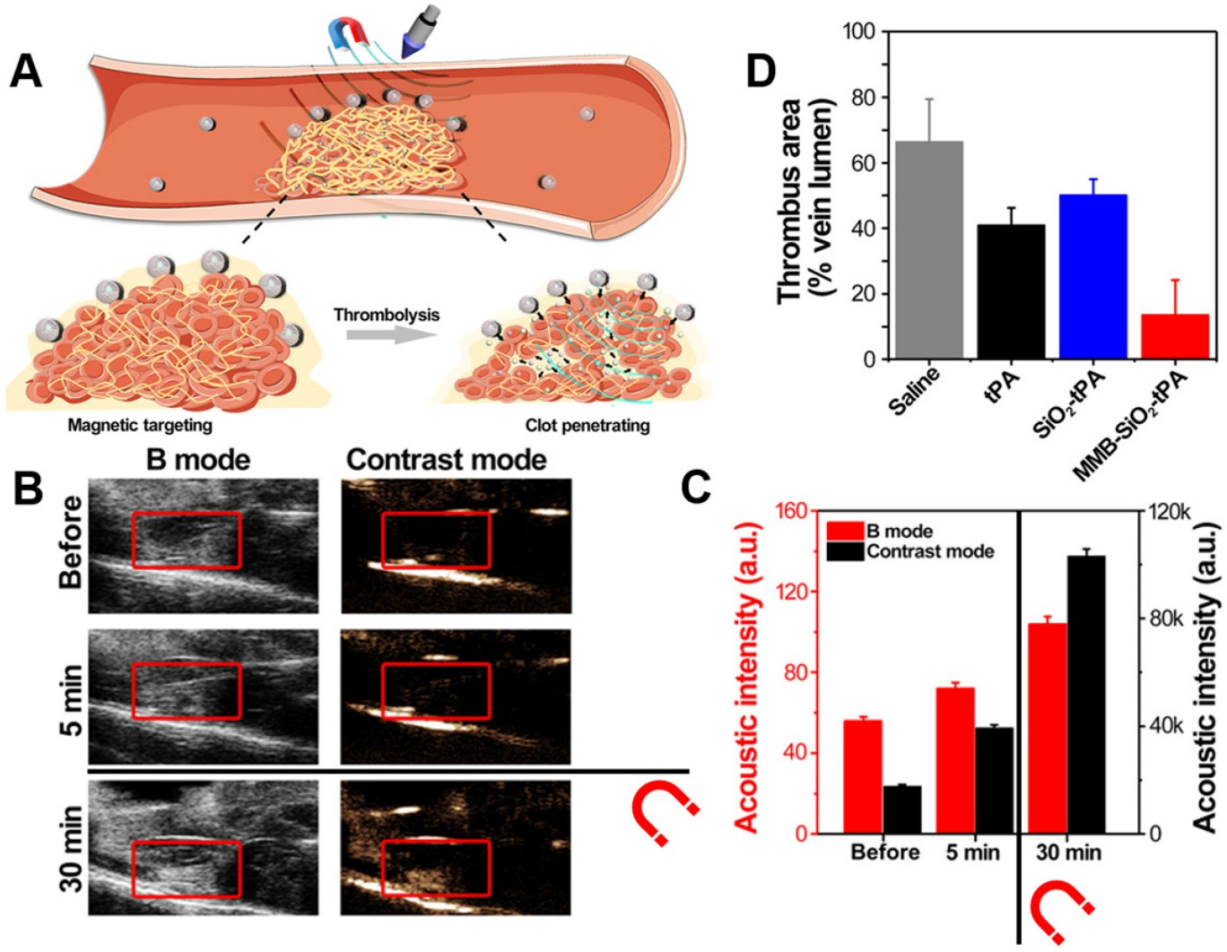
** Magnetically targeted, LIFU responsive silica-shelled microbubbles for accelerated thrombolysis. A.** Schematic representation of magnetic targeting and the LIFU responsive behaviour of the microbubbles. **B.** US imaging of FeCl_3_-induced venous thrombosis before, 5, and 30 minutes after administration of the microbubbles. Magnetic targeting to the thrombus was commenced after 5 minutes as indicated. **C.** Quantification of the acoustic intensity observed during US imaging. Data are shown as mean ± SD (n = 3). **D.** Thrombus area in a FeCl_3_-induced venous thrombosis mouse model after treatment with saline, tissue plasminogen activator (tPA), non-magnetic silica-shelled microbubbles (SiO_2_-tPA), and magnetic silica-shelled microbubbles (MMB-SiO_2_-tPA). Data are shown as mean ± SD (n = 4). Adapted with permission from Wang *et al.* 39, Copyright 2020 AAAS.

Wang *et al.* exploited this by producing a magnetically targeted theranostic system based on microbubbles shelled by IONPs and silica nanoparticles. (**Figure 8A**) 39. They tested this system in a ferric chloride-induced femoral vein thrombosis mouse model, with promising results. Upon placement of a magnet on the site of embolism, the particles accumulated there, enabling higher contrast US imaging (**Figure 8B,C**). Additionally, the targeting increased the system's thrombolytic potential *in vivo* compared to the non-targeted equivalent (**Figure 8D**). When compared to the other targeting approaches discussed in this review, this system also benefits from a more simplistic method of production. The IONPs utilised for targeting are easier to produce and incorporate compared to the preparation of ligands and their conjugation onto nanoparticles, increasing the relative cost-effectiveness of this system. Additionally, the functionality of IONPs in MRI 35, MPI 68, and even magnetically-induced hyperthermia 70 may enable the production of multifunctional, highly sophisticated, yet easy to fabricate theranostic systems. Though this approach appears highly promising, caution should also be taken; magnetic particles have also been designed specifically to cause vessel occlusion through magnetically-induced aggregation 99. Although this vessel occlusion has not yet been reported with nanoparticles designed for the treatment of thrombosis, this may be a possibility that should be investigated to avoid adverse effects. Additionally, active guidance as applied here is limited by the need for input from a trained professional, thus reducing its clinical applicability.

Hence, there is not a single targeting approach without limitations, explaining why clinical adoption of targeted theranostics for thrombosis is yet to occur. It appears particularly challenging to find a targeting approach that combines both a high affinity while retaining favourable cost-effectiveness and ease of use. Hence, further research is required to find such an approach, where it is expected that advancements in this area may aid in elevating the field of theranostic nanoparticles for thrombosis towards clinical use.

## Early detection of thrombosis

Besides use in the delivery of therapeutics, the above-mentioned approaches for active targeting and responsive behaviour may also be applied to the early detection of thrombogenesis, which is currently highly challenging. Clinical guidelines dictate early diagnosis of thrombosis should be attempted via diagnostic imaging or D-dimer tests depending on the risk, though both approaches exhibit limitations 122. For instance, conventionally used imaging techniques such as US, CT, and MRI rely on the detection of an obstruction of blood flow, or direct imaging of the dense thrombus, as was discussed previously. Therefore, they lack the ability to accurately identify smaller and non-occluding thrombi. Alternatively, D-dimer tests are used to indicate activation of the body's natural fibrinolytic response to thrombogenesis, which results in elevated D-dimer levels. However, these tests provide no information on the location of the thrombus, and wide variations in the type and operating characteristics of these assays complicate their use and hinder cross-test extrapolation 122. Thus, the early detection and subsequent prevention of acute thrombotic events remain challenging. Hence, the development of theranostic nanoparticles that can detect young, small thrombi and prevent their progression to larger, unstable clots may be highly beneficial in the prevention of stroke, myocardial infarction, and pulmonary embolisms.

Historically, simple imaging probes have been investigated for this purpose, with a focus on the application of thrombus-targeting moieties, enabling the accumulation of the imaging probe at the site of small thrombi which are otherwise impossible to detect. A rather simple example of this is EP-2104R, which consists of a fibrin-targeting peptide coupled to 4 gadolinium chelates 123. This approach enabled the identification of thrombi in MRI not visible in contrast-free screening in phase II clinical trials, thus showing great promise for the earlier detection of thrombi compared to conventional imaging 124. However, such a simple probe lacks the ability to prevent the development of blood clots once detected. Therefore, the application of theranostic nanoparticles may be of high clinical relevance in this area, where it may improve pharmacokinetics, and enable simultaneous imaging and thrombosis prevention.

This area of interest was identified by Guo *et al.*, where they highlighted two theranostics systems as potentially capable of the early detection of thrombosis 32, 33. To do so, these systems are decorated with cRGD or EWVDV to enable an association with activated platelets as present in the early thrombogenic environment. However, both systems were designed to treat acute thromboembolisms, as they exert clot degradation through the delivery of a thrombolytic (rtPA) or microbubbles for US-induced cavitation 32, 33. This approach is not very relevant for the treatment of early thrombosis, where delivery of an antithrombotic to prevent progression of thrombogenesis may be preferable. For instance, Lee *et al.* developed a theranostic system for the delivery of aspirin, using GPRPP as a targeting ligand 76. Though its ability to detect early-stage thrombosis has not been tested, it is expected that such a system should be capable of doing so as it displayed adequate affinity to thrombi. Unfortunately, the system does rely on optical imaging, thus the development of systems relying on more clinically relevant imaging techniques would be beneficial.

Finally, one promising approach yet to be applied to theranostic nanoparticles is the early detection of thrombosis through responsive imaging as stimulated by thrombosis biomarkers. Similar to the responsive systems previously described, sensitivity to thrombin may be incorporated through the use of a thrombin-sensitive peptide. Thus, by conjugating a fluorescent dye through this thrombin-sensitive peptide to fluorescence-quenching nanoparticles, nanoprobes that only display a fluorescence signal in the presence of thrombin have been created 125, 126. Unfortunately, such an approach is limited by the low depth of penetration of optical imaging, thus nanoprobes with thrombin-responsive US 127 and X-ray-excited luminescence (X-ray photon in, optical photon out) 128 have also been developed. Though these systems did not deliver therapeutics and therefore cannot be classified as theranostics, the use of such an approach in theranostics may be highly beneficial. Particularly interesting may be the development of nanoparticles where thrombin can activate both imaging and drug release, creating a sophisticated solution for the detection and treatment of early thrombosis. However, the complex synthesis of such a sophisticated system may be considered a hurdle. The need to incorporate two imaging moieties to enable both thrombin-responsive imaging as well as non-responsive biodistribution tracking exacerbates this issue. This, once again, shows that research into systems that are both sophisticated and easy to manufacture is required to maximise their clinical potential.

## Pre-clinical assessment of theranostic nanoparticle systems

Accurate and reliable pre-clinical testing to assess the potential of these theranostic nanoparticles is vital for the successful elevation towards clinical application. Recently, there has been a push throughout medical research towards the decreased use of *in vivo* models out of ethical considerations, and a lack of predictive value of animal models for successful clinical translation. Thus, research into more predictive *in vitro* models has increased drastically. This includes the development of better cell models which can predict physiological response more accurately, as well as the introduction of microfluidic chip models. This also applies to thrombosis, with the development of a thrombosis-on-a-chip model for instance 129, 130. However, *in vivo* models are also still widely used, as they provide superior ease-of-use and predictability while maintaining low costs 131.

*In vitro* assessment of antithrombotics is generally performed utilising relatively simple blood clot models. In such models, thrombin is added to whole blood, resulting in thrombogenesis. Breakdown of the clot as a result of the antithrombotic can then be measured easily using optical imaging, giving an accurate indication of the thrombolytic potential of the system 132. As whole blood is generally easy to obtain from small animals, and is a very accurate representation of the pathophysiology, this model is widely used to assess nanoparticle systems prior to commencing *in vivo* studies 55. Additionally, such models can be used to assess the targeting affinity of the nanoparticles to whole blood clots by simply determining their association with the thrombi after washing 42, 55. Targeting of specific parts of thrombi can be tested utilising flow cytometry models, which are frequently used to determine affinity for activated platelets 42, 132. All these models are easy to set up and reasonably accurate, though they fail to recapitulate some important physiological aspects of thromboembolisms. For instance, these systems cannot mimic the mononuclear phagocyte system, which impacts the pharmacokinetics of nanoparticles 133. Perhaps more importantly, however, is the omission of the complexity and interactions of blood vessel walls in these models, which plays a large part in thrombogenesis 131.

To enable more sophisticated *in vitro* models with improved physiological mimicking, the development of so-called organ-on-a-chip models has gathered significant interest over the last decade. Such models involve the production of a set of microchannels, where (human) cells can be cultured followed by perfusion with a fluid 134. These microfluidic systems can mimic and predict biological and biochemical interactions close to *in vivo* models, while retaining the benefits of *in vitro* models, including easier monitoring and improved control over biological conditions. Hence, such systems have also been applied to mimic thrombosis by producing blood vessel-simulating models, resulting in thrombosis-on-a-chip models 134. Of particular interest is the excellent control over shear in these microfluidic models, as this is a major factor in thromboembolisms 135.

**Figure 9:**
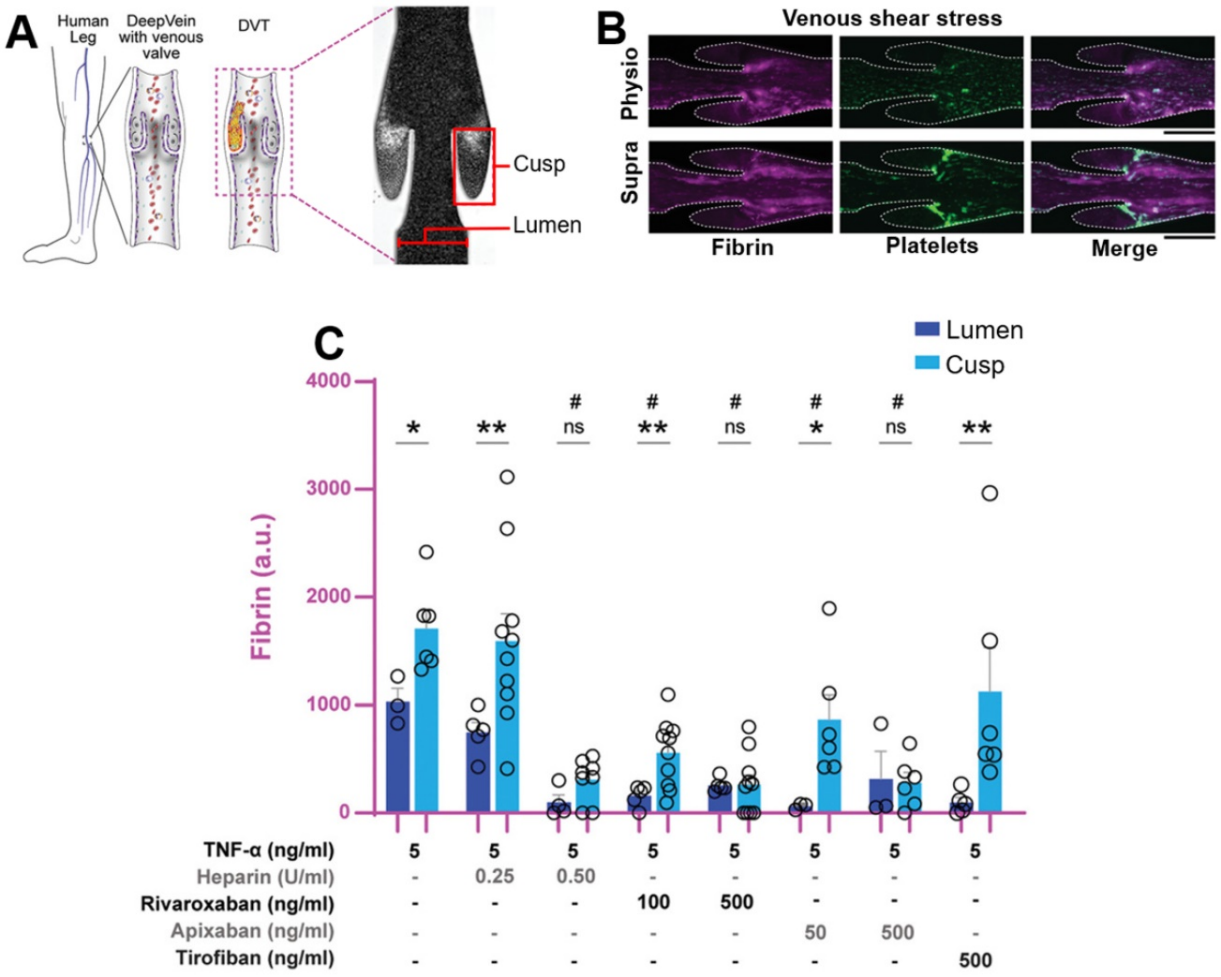
** A DVT-on-a-chip microfluidic model was developed to study the interaction between thrombi and venous valves. A.** Illustration of normal human vein versus vein with thrombus formation, and comparison with brightfield microscopic snapshot of a venous valve in the DVT-on-a-chip microfluidic model with identification of the lumen and cusp of the valve. **B.** Fluorescence micrographs of fibrin (left), platelets (middle), and merge (right) in a venous valve under normal shear (physio) and pathological shear (supra, ≈17.5 dynes ^cm-2^). **C.** Graph showing fibrin formation in the lumen and cusp of venous valves in the DVT-on-a-chip model upon treatment with TNF-α (pro-coagulant) and perfusion with whole blood containing an anticoagulant (Heparin, Rivaroxaban, or Apixaban) or antiplatelet (Tirofiban). Data points indicate independent experiments, with bars displaying mean ± SD. Lumen versus cusp: *, p<0.05, **, p<0.01; Untreated versus treated cusp: #, p<0.05. Adapted with permission from Jain *et al.* 137, copyright 2020 Wiley.

Several studies have assessed the thrombolytic potential of nanoparticle systems by utilising relatively simple microfluidic models to mimic microvessels 129, 130. More sophisticated models have also been established, including a microfluidic model of pulmonary embolisms 136, and a highly impressive model of DVT provided by Pandian *et al.* (**Figure 9**) 137. This microfluidic model enables the investigation of the highly intricate interaction between thrombi and venous valves 137. The effect of elevated shear stress as commonly observed in thrombosis could also be determined, showing increased deposition of platelets in the cusp as was expected, but also decreased deposition of fibrin in the lumen - an unexpected effect (**Figure 9B**). Such findings would have been very difficult to obtain if an *in vivo* model was utilised instead, indicating the benefit of microfluidic technology. Additionally, the antithrombotic potential of drug treatment could also easily be measured, again resulting in observations hard to make with *in vivo* models (**Figure 9C**) 137. This showed that traditional dosage regimes of anticoagulants are insufficient, requiring a significant increase in dose to adequately prevent thrombus formation in venous valve cusps. Additionally, the ineffectiveness of antiplatelet agents in the prevention of DVT was shown, as Tirofiban had little effect on thrombus formation in valve cusps due to the lack of platelets in this location 137.

However, these microfluidic organ-on-a-chip models also exhibit some limitations, as they are quite complex to design and cannot achieve the accuracy of physiological representation provided by *in vivo* studies 131, 134, 137. Hence, animal models are still widely used to determine the clinical potential of theranostic nanoparticles for thrombosis. Due to their inherent imaging capabilities, *in vivo* monitoring of theranostics is particularly easy and valuable. Specifically, a FeCl_3_-induced thrombosis model is so common as it is easy to establish and is robust 131. In this model, a blood vessel is exposed through an incision, after which a FeCl_3_-soaked filter paper is placed on the vessel, resulting in thrombosis 138. Interestingly, the mechanism through which this approach produces thrombi was poorly understood until a microfluidic model was developed, again indicating the benefits of deploying organ-on-a-chip models to understand biological interactions 139. Regardless, the ferric chloride thrombosis model is used frequently due to its superior accuracy of blood-to-vessel wall interactions 131.

**Figure 10:**
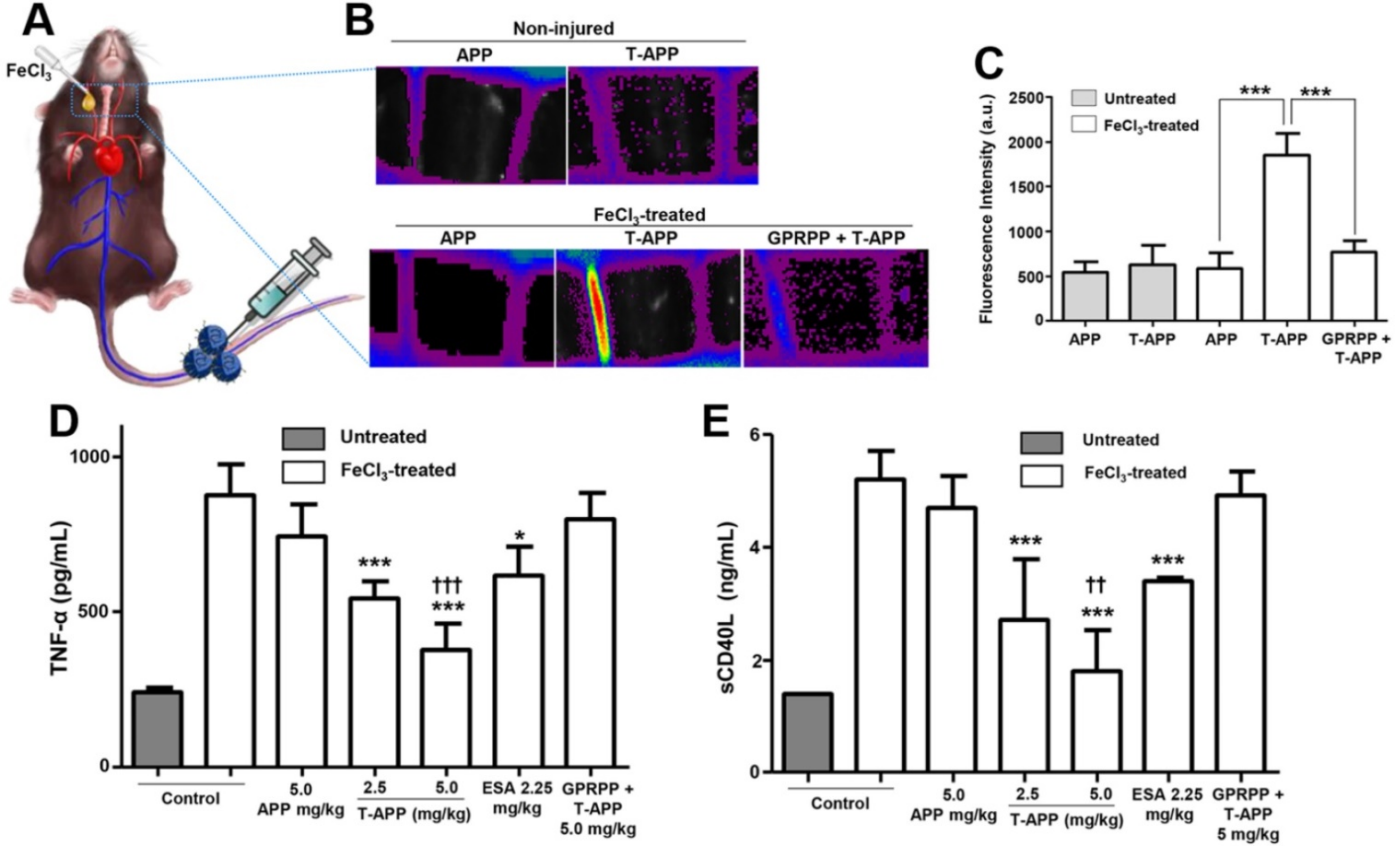
** Evaluation of thrombus targeting aspirin theranostic nanoparticles in a mouse model of FeCl_3_-treated carotid arterial thrombosis. A.** Schematic illustration of the mouse model of FeCl_3_-treated carotid arterial thrombosis. **B.** Fluorescence images of the carotid artery of mice administered with non-targeted (APP), targeted aspirin theranostic nanoparticles (T-APP), and/or free targeting ligand (GPRPP) before and after injury with FeCl_3_ to induce thrombosis. **C.** Quantification of the fluorescence intensity observed in **B**. Values are mean ± SD (n = 4). ***, p < 0.001. **D.** Suppression of TNF-α by APP, T-APP, and known antiplatelet ethyl salicylate (ESA) to indicate anti-inflammatory efficacy. **E.** Suppression of sCD40L by APP, T-APP, and ESA to indicate antiplatelet efficacy. All values are mean ± SD (n = 4). *p < 0.05, ***p < 0.001 relative to control. ^††^p < 0.01, ^†††^p < 0.001 relative to equivalent ESA. Adapted with permission from Lee *et al.* 76, copyright 2019 Elsevier.

Hence, many of the theranostic nanoparticles for thrombosis discussed here have been assessed through this model, focussing mostly on arterial thrombosis (**Figure 4, 6**). Lee *et al.* showed a particularly impressive example of the power of such a model 76. Here, the use of a mouse model of FeCl_3_-induced carotid arterial thrombosis (**Figure 10A**) enabled them to demonstrate the effect of active targeting on the accumulation of the theranostic at the site of thrombosis. Because the carotid arteries are exposed in this model, fluorescence microscopy can be used to measure the presence of the theranostic (**Figure 10B**). Subsequent quantitative analysis of said images enables the statistical significance of the results to be determined (**Figure 10C**). Additionally, the anticoagulant properties of the developed theranostic agent could also be accurately quantified by measuring the presence of pro-thrombotic biomarkers. This includes tumour necrosis factor-alpha (TNF-α) (**Figure 10D**), which is an indicator of inflammation in the endothelial cells, and soluble CD40L ligand (sCD40L) (**Figure 10E**), which is produced by activated platelets 76. Hence, Lee *et al.* could show their theranostic system reduced these biomarkers more than an equivalent dose of aspirin, indicating significantly higher anti-inflammatory and antiplatelet efficacy. As these biomarkers result from complex biological processes, such results may not have been as accurate if *in vitro* models were utilised instead. Mimicking of human physiology could be improved even further by using large animal models, to which end a canine model has also been established 140. However, due to the prohibitive costs associated with such a model, it is thought that FeCl_3_-induced small animal thrombosis models will remain the predominant mode of assessment in this field for some time to come.

Besides these FeCl_3_-induced thrombosis models, multiple other models for more specific specialisations have also been developed. This includes models of thrombosis as induced by other stimulants, including flow restrictions and collagen 141, 142. Models for specific thrombotic events, including stroke and pulmonary embolisms, have also been established, where such advanced models are highly beneficial for the in-depth study of thrombosis and its treatments 42, 143. As previously mentioned, the design of theranostic nanoparticles should be tailored for their purpose, thus models for thrombosis other than arterial thrombosis must be established. However, the greatest benefit to the field of thrombosis treatment (including theranostics) would arguably be the widespread adoption of a standardised *in vivo* model. To this end, multiple groups have worked towards the standardisation of the ferric chloride model, which would be the ideal candidate for such a purpose due to its simplicity, accessibility, and reproducibility 138, 144. Adoption of such a standardised model would enable comparison of treatment approaches, as the variation in experimental conditions is reduced. Theranostic nanoparticles in particular can benefit from standardisation, as imaging capabilities, responsive behaviour and targeting can all be compared directly. Hence, promising approaches may be identified much more easily, aiding greatly in the elevation of theranostic nanoparticles for thrombosis towards clinical adoption.

## Perspective

Theranostic nanoparticles are a highly promising approach towards resolving the issues currently related to the diagnosis and treatment of thrombosis. By simultaneously delivering imaging and therapeutic moieties, time to diagnosis and subsequent treatment of thromboembolisms may be quickened, improving morbidity and mortality related to blood clotting events. Nanoparticles enable highly efficient delivery of antithrombotics and can reduce complications such as bleeding, which are currently frequently observed. Current theranostic nanoparticles have shown great promise in these areas, improving efficacy while reducing side effects. However, clinical translation of these theranostic nanoparticles for thrombosis has been limited. Thus, current research focuses on addressing some of these issues, potentially ushering the field of theranostic nanoparticles towards clinical use for the diagnosis and treatment of thrombosis.

This review has assessed the current theranostic nanoparticles for thrombosis according to four criteria that require optimisation to maximise the clinical potential of efficacious theranostic nanoparticles. These criteria are; (1) safety; (2) simplicity and rapidity; (3) applicability and availability; (4) cost-effectiveness. Clinical translation of current theranostic nanoparticles for thrombosis has been limited due to the shortcomings of each system in at least one of these areas. Based on these shortcomings, recommendations on approaches towards the design of theranostic nanoparticles can be made.

First, and of the greatest concern, is the safety of the theranostic nanoparticles. To ensure clinical uptake, these nanocarriers must not pose any additional risk of side effects. Hence, theranostic nanoparticles must be designed with biodegradability and low toxicity in mind. This primarily encourages the use of nanomaterials that are biodegradable, non-immunogenic, and non-toxic at the intended dosage. Several common approaches fail in this aspect, where the materials and dosage used during *in vitro* and *in vivo* studies would likely result in serious complications if used clinically. Hence, further research into the development of highly biocompatible and degradable nanoparticles is paramount to ensuring the safety profile of said systems when applied clinically. Furthermore, loading of both the imaging modality and therapeutic must be optimised to ensure both are present at relevant concentrations, as to avoid diagnostic/therapeutic dose discrepancy and subsequent toxicity. Finally, the safety of the imaging technique employed should also be considered, where particularly repeated exposure to ionising radiation should be minimised.

Specific to theranostic nanoparticles designed for the management of thrombosis is the need to mitigate haemorrhagic risk. Due to the severe bleeding side effects commonly experienced with thrombolytics and anticoagulants, theranostic nanoparticles must limit their release away from the thrombus. However, some particles are lacking in this aspect, where they are mostly limited by either non-specific release of the payload or surface loading of the therapeutic onto the nanoparticle. Both of these aspects allow interaction of the therapeutic with tissue at sites other than the thrombus, resulting in potential bleeding effects. Hence, there is a need to design particles with the therapeutic loaded in the core and specific delivery to the thrombus. As identified in this review, both active targeting and responsive release are promising strategies to improve specificity and subsequent safety of theranostics. High specificity and affinity of the targeting ligand can enhance drug delivery to the thrombus while limiting accumulation in non-target tissues. The specificity of responsive release is closely related to the choice of stimulus, where guided external stimuli and thrombin were identified as resulting in the most specific release. Although a very fast release could be very efficacious, release rates should be managed to prevent clot fragmentation and subsequent blockage of smaller arteries.

The simplicity and speed of the system are particularly vital for the treatment of severe acute thromboembolisms, as tissue damage is rapid upon occlusion of a blood vessel. Some theranostic systems lack in their simplicity of use, particularly due to the need for input of an external stimulus by a trained professional. Hence, it is recommended that responsive drug released is induced by internal stimuli and that targeting of the theranostic to the site of thrombus is performed through targeting ligands rather than magnetic guidance. The rapidity of the system is particularly important for the treatment of acute thromboembolisms, and is related to several aspects of its design. Hence, the time to diagnosis should be minimal, and the breakdown of the clot should be fast. To maximise the speed of diagnosis, an imaging technique with rapid imaging and post-processing should be utilised. Thus, the use of MRI in emergency situations is discouraged, where faster imaging techniques such as US, PAI, SPECT/CT, or PET/CT are preferable. The speed accumulation of the theranostic at the site of embolism is also vital to expedite treatment, hence the use of targeting ligands with high affinity for their target (such as single-chain antibodies) is recommended. Furthermore, due to the dense structure of thrombi, enhancing the penetration into the clot may also be beneficial to ensuring rapid and effective treatment. To this end, theranostic systems have thus far utilised CPPs and microbubbles, though nanomotors may also be of interest as an emerging strategy. These nanomotors can be propelled magnetically or through stimulation with NIR, resulting in penetration of the clot 130, 145. Hence, such an approach could be highly beneficial if applied in a theranostic system. Finally, the responsive release profile of thrombolytic should also be rapid to ensure the time to the breakdown of the clot is minimised. However, as mentioned before, the release profile should be carefully managed to prevent unwanted side effects, thus the speed of release should be managed with both safety and rapidity in mind.

The applicability and availability of the theranostic nanoparticles are also of great importance to ensure their clinical application. The applicability of the theranostic relies most prominently on the type of thrombosis, where characteristics such as size, age, location, and platelet content all affect the efficacy of the treatment. Hence, theranostic nanoparticles should be designed with a particular application in mind. This includes the choice of responsiveness, targeting ligand, and even therapeutics. The choice of imaging technique should also be considered, where less acutely dangerous types of thrombosis (such as DVT) can benefit from slower but safer and more accurate diagnostic imaging for instance. The availability of the imaging technique is also an important factor, where the clinical application of a theranostic agent reliant on expensive and unavailable equipment will likely be limited.

Finally, to ensure clinical uptake of theranostic nanoparticles, their cost-effectiveness should also be considered. Theranostics are already somewhat complex due to the need to incorporate both an imaging moiety and a therapeutic, thus they should be designed with ease of GMP production and cost in mind. Many of the current theranostic nanoparticles for thrombosis fail in this aspect, where complex synthesis is utilised to produce highly sophisticated systems. To retain the sophisticated functionality of these systems while reducing their complexity this review recommends the use of multifunctional materials. For instance, due to their magnetic properties, IONPs not only function as a nanoparticle framework, but have also been used for magnetic targeting, MRI, MPI, and magnetically-induced hyperthermia. Hence, surface loading of IONPs or incorporation of IONPs into other nanoparticles enables the production of theranostics with multiple functionalities without the need for the conjugation of several ligands, reducing the complexity of synthesis and thus increasing the cost-effectiveness. However, there are also other ways to reduce the complexity of synthesis, such as the use of less complex ligands for targeting and responsive release. Hence, more research would need to be performed into the development of simple theranostic nanoparticles to increase their commercial potential.

Many design aspects affect the clinical potential of theranostic nanoparticles for thrombosis. As of yet, no one system has met all four criteria, thus clinical translation thus far has not been achieved. However, as shown throughout this review, theranostic nanoparticles have been highly efficient at the diagnosis, prevention, and treatment of thrombosis in pre-clinical models, while reducing side effects significantly. It is expected that with more research in this area, the development of such systems with higher commercial and translational potential could enable clinical application, and subsequently reduce morbidity and mortality related to thrombosis.

## Figures and Tables

**Table 1:** Overview of all reported theranostic nanoparticles for thrombosis sorted by year of publication, with identification of the nanoparticle type, imaging technique, and moiety, their mode of therapeutic action, responsiveness, and targeting utilised

Year	Nanoparticle Type	Imaging Technique	Imaging Moiety	Therapeutic Action	Responsiveness	Targeting	Reference
2021	AuNR	Optical/CT	Dye (ICG)/ AuNR	Urokinase (thrombolytic), Fucoidan (anticoagulant)	-	Fucoidan	47
2021	Polymeric micelle	PAI	Dye (IR780)	Lumbrokinase (thrombolytic)	-	FXIII peptide	55
2020	PLGA polymersome with platelet membrane	Optical	Dye (FITC)	rtPA (thrombolytic)	-	Platelet membrane	42
2020	Nanodroplet	US	Microbubble	Microbubble	LIFU/thrombin	ACPP/FTP	40
2020	PLGA shell-coated nanodroplet	Optical/PAI	Microbubble/ Dye (ICG)	Microbubble	LIFU	cRGD	41
2020	Carbon dot	Optical	Carbon dot	Urokinase (thrombolytic)	-	-	146
2020	IONPs and porous silica microbubbles	US	Microbubble	tPa (thrombolytic)	LIFU	Magnetic	39
2019	Aspirin-polymer	Optical	Dye (IR780)	Aspirin (antiplatelet)	H_2_O_2_	GPRPP	76
2019	PLGA shell-coated nanodroplet	MRI/PAI	IONPs	Microbubbles	LIFU	CREKA	34
2018	Maltodextrin	US/PAI	Microbubble/ Dye (IR780)	HBA (anti-inflammatory)	H_2_O_2_	GPRPP	53
2018	Erythrocyte microvesicle	Optical	Dye (ICG)	tPA (thrombolytic)	-	Erythrocyte	43
2017	Polymersome	Optical/PAI	Dye (IR820)	HBA (anti-inflammatory)	H_2_O_2_	CREKA	54
2017	PLGA shell-coated nanodroplet	MRI/optical/PAI	IONPs	Microbubble	LIFU	EWVDV	32
2017	IONPs	MRI	IONPs	Heparin (anticoagulant)	-	-	31
2016	Microbubble	US	Microbubble	scuPA (thrombolytic)	-	scFv_SCE5_	38
2015	macrophage microvesicle	MRI	IONPs	tPA (thrombolytic)	-	Magnetic	35
2014	PLGA polymersome	MRI	IONPs	rtPA (thrombolytic)	-	cRGD	33
2014	PFC nanodroplet	MRI	^19^F	PPACK (antithrombotic)	-	PPACK	57
2012	IONPs	MRI/optical	IONPs	tPA (thrombolytic)	-	FXIII peptide	30
2011	PFC nanodroplet	MRI	^19^F	PPACK (antithrombotic)	-	PPACK	58

**Table 2:** Systematic comparison of imaging modalities with applications in diagnosis of thrombosis

Modality	Resolution	Sensitivity	Depth	Clinical availability	Contrast agents	Drawbacks
US	Moderate	Moderate	Limited by overlying structures	High	Microbubbles	Reliance on operator skill, lower resolution and sensitivity than PAI
CT	Moderately high	Moderate	Unlimited	High	Iodinated contrast media	Radiation, toxicity of contrast agent
MRI	High	Low	Unlimited	Moderate	IONPs, Gd^3+^, ^19^F, CEST agents	Cost, time of imaging, abundant contraindications
Optical	High	High	<1 cm	Limited	Fluorescent dyes	*In vivo* use is limited by the depth of penetration
PAI	Very high	Moderately high	7 cm	Emerging	NIRF dyes, gold nanoparticles, carbon nanotubes	Limited depth of penetration
SPECT	Low	Very high	Unlimited	High	Gamma emitting radioisotopes (^99^Tc, ^123^I, ^201^Tl)	Radiation, no structural information
PET	Low	Very high	Unlimited	Moderate	Positron emitting radioisotopes (^18^F, ^64^Cu, ^8^Ga)	Radiation, no structural information, radioisotopes hard to use
MPI	High	High	Unlimited	Emerging	IONPs	No structural information

## Abbreviations

APP: aspirin nanoparticle
AuNP: gold nanoparticle
AuNR: gold nanorod
CEUS: contrast enhanced ultrasound
COVID-19: coronavirus disease 2019
CPP: cell-penetrating peptide
CT: computed tomography
CVD: cardiovascular disease
DVT: deep vein thrombosis
ESA: ethyl salicylate
FTP: fibrin targeting peptide
Fu: Fucoidan
IONP: iron oxide nanoparticle
LIFU: low intensity focussed ultrasound
MPI: magnetic particle imaging
MRI: magnetic resonance imaging
NP: nanoparticle
PAI: photoacoustic imaging
PE: pulmonary embolism
PET: positron emission tomography
PFH: perfluorohexane
PLGA: poly(lactic-co-glycolic acid)
PTCA: percutaneous transluminal coronary angioplasty
RBC: red blood cell
ROS: reactive oxygen species
rtPA: recombinant tissue plasminogen activator
sCD40L: soluble CD40 ligand
scFv: single chain antibody
scuPA: single-chain urokinase
SD: standard deviation
SPECT: single-photon emission computed tomography
sPLA_2_: secreted phospholipase A_2_
TF: tissue factor
TI: tirofiban
TNF-α: tumour necrosis factor-alpha
tPA: tissue plasminogen activator
UK: urokinase
US: ultrasound
vWF: von Willebrand factor
